# On the Utmost Importance of the Basis Set Choice for the Calculations of the Relativistic Corrections to NMR Shielding Constants

**DOI:** 10.3390/ijms24076231

**Published:** 2023-03-25

**Authors:** Irina L. Rusakova, Yuriy Yu. Rusakov

**Affiliations:** A. E. Favorsky Irkutsk Institute of Chemistry, Siberian Branch of the Russian Academy of Sciences, Favorsky St. 1, 664033 Irkutsk, Russia; rusakov82@mail.ru

**Keywords:** relativistic correction, HALA effect, HAHA effect, basis set, NMR shielding constant, NMR chemical shift

## Abstract

The investigation of the sensitivity of the relativistic corrections to the NMR shielding constants (σ) to the configuration of angular spaces of the basis sets used on the atoms of interest was carried out within the four-component density functional theory (DFT). Both types of relativistic effects were considered, namely the so-called heavy atom on light atom and heavy atom on heavy atom effects, though the main attention was paid to the former. As a main result, it was found that the dependence of the relativistic corrections to σ of light nuclei (exemplified here by ^1^H and ^13^C) located in close vicinity to a heavy atom (exemplified here by In, Sn, Sb, Te, and I) on the basis set used on the light spectator atom was very much in common with that of the Fermi-contact contribution to the corresponding nonrelativistic spin-spin coupling constant (*J*). In general, it has been shown that the nonrelativistic *J*-oriented and σ-oriented basis sets, artificially saturated in the tight *s*-region, provided much better accuracy than the standard nonrelativistic σ-oriented basis sets when calculating the relativistic corrections to the NMR shielding constants of light nuclei at the relativistic four-component level of the DFT theory.

## 1. Introduction

Nuclear Magnetic Resonance (NMR) spectroscopy represents one of the most powerful tools for chemical structure studies. It has become common practice to combine high-quality quantum chemical modeling of spectra with NMR experiments. In this respect, much effort has been put into the development of accurate and efficient quantum chemical computational protocols for NMR parameters [1,2,3], and special attention has been paid to the effects of special relativity manifesting in the NMR parameters of compounds containing heavy elements [4,5,6,7,8,9,10,11,12]. In particular, these may have a significant impact not only on the NMR shielding constants of heavy nuclei [8,13,14,15,16], but also on those of the light nuclei [17,18,19,20,21,22,23]. The former type of relativistic effects is known as the HAHA effect [24,25,26,27], which is the heavy atom effect on the NMR shielding constant of the heavy atom itself. The latter type is called the HALA effect [28,29,30,31], i.e., the heavy atom effect on the NMR shielding constant of the light atom. Broadly speaking, the accuracy of the calculation of both types of relativistic effects depends on three main factors: the level of taking into account the effects of electron correlation, the level of relativistic Hamiltonian applied (inferring the level of accounting for the relativistic effects), and the quality of the basis set used.

The first two factors can be attributed to the level of theory. This has made stable progress in the last decades; many salient reviews were published on the relativistic quantum-chemical methods for the calculation of NMR parameters [32,33,34,35,36,37]. As far as we are aware of, only two methods for the calculation of NMR shielding constants were formulated in terms of the relativistic quantum theory and are now available in modern quantum chemical packages. These are the random phase approximation (RPA) [38] and the density functional theory (DFT) [39,40]. Accordingly, their most accurate relativistic analogues are the four-component random phase approximation (4c-RPA) [32,41,42] and the four-component density functional theory (4c-DFT) [43]. The 4c-RPA has been extensively used in the calculations of shielding constants for a long time [8,44,45], though it has not received as much popularity as the 4c-DFT method due to omitting the electron correlation effects. The 4c-DFT method is capable of providing fairly accurate results because it simultaneously accounts for the relativistic effects within a four-component Dirac-Kohn-Sham Hamiltonian along with the electron correlation effects via the exchange-correlation potential, and, at the same time, its computational cost is rather moderate, being similar to that of the 4c-RPA approach (the two-electron molecular integral transformation requires *O*(*N*^5^) operations [46]). There is also another very accurate, highly correlated four-component method that is called the second-order polarization propagator approach (4c-SOPPA) [47]. This method represents a real breakthrough in the relativistic computational NMR field; however, it has not been implemented in standard program packages for now. As a result, for the time being, the only way to account for both the relativistic and electron correlation effects is to use the relativistic DFT formalism, which has given rise to the most popular relativistic four-component DFT method for the calculation of the NMR shielding constants and the relativistic corrections to them.

The third factor that heavily affects the calculation of NMR shielding constants (σ) at the relativistic four-component level of theory is the choice of the basis sets used for the description of large spinor components. This factor has not been investigated systematically yet. Even more so, a completely uninvestigated issue is what kind of basis sets are suitable for the calculation of the relativistic corrections to the shielding constants in the systems containing heavy atoms. In the meantime, accurate calculations of the relativistic corrections to NMR shielding constants at the four-component DFT level of theory represent a great importance when one adds the relativistic corrections to the basic values calculated within the high-quality nonrelativistic ab initio correlated approaches, like CCSD [48] or CCSDT [49,50]. In this case, if the relativistic corrections were calculated incorrectly, in particular due to the inappropriateness of the basis sets used, the total values may fall out far beyond reasonable limits, which makes all these highly demanding calculations in vain. In this way, the main goal of this study is to find out what types of basis sets are suitable for the calculation of the relativistic corrections to the shielding constants of light nuclei in systems containing heavy atoms (the HALA effect). At the same time, given that the choice of basis sets for the calculation of the shielding constants of heavy atoms (the HAHA effect) is of no less importance, this issue has also received a notable amount of attention. We also intended to find out whether there is a coherence in the dependences of the HALA effect on the shielding constants of light atoms and the relativistic shielding constants of light atoms per se, depending on the basis set used.

At this point, a reasonable question that can be raised is: why should one take special care of the large component basis set when calculating the relativistic corrections to the NMR shielding constants at the four-component level of theory? Why not take some standard large energy-optimized basis sets that presumably should be sufficient to provide a reliable description of any molecular property at the four-component level of theory, including the NMR shielding constants and the relativistic corrections to them? The answer to this question lies in the high computational demands of the four-component methods. Even for the nonrelativistic level of the theory, it is well known that standard energy-optimized basis sets are not effective for the calculation of the second- and higher-order molecular properties, like NMR shielding [51] and spin-spin coupling constants (SSCCs) [52,53,54]. This is because such basis sets do not usually provide an appropriate representation of the molecular orbitals in the desired areas and provide slow convergence towards the complete basis set (CBS) limit [55]. As a consequence, to achieve values close to the CBS limit within a particular method, one is forced to resort to rather large nonspecialized basis sets [2], resulting in high computational demands. For the four-component level of the electronic theory, this issue becomes much more crucial because the computational requirements for a particular relativistic four-component computational method exceed those of the nonrelativistic analog by several times of magnitude only due to the fact that the equations are formulated within a four-spinor functional space [56,57,58].

In the nonrelativistic level of theory, many efforts have been made to develop efficient specialized basis sets for NMR properties in order to decrease the computational costs of their quantum-chemical calculations. In particular, there were many specialized basis sets introduced for spin-spin coupling constants (*J*-oriented basis sets) [52,59,60,61,62,63,64,65,66,67,68,69,70,71,72,73] and, to a lesser extent, for chemical shifts or shielding constants (σ-oriented basis sets) [51,74,75,76]. At the same time, for the relativistic level of theory, the issue of specialized basis sets for NMR parameters still remains open. Namely, for the relativistic calculations of spin-spin coupling constants, there were only the TZ2P-J and QZ4P-J STO basis sets developed within the ADF program package [77,78]. For the relativistic calculations of shielding constants, there has only been one series of specialized basis sets introduced recently for almost all nuclei (H-Rn, La-Lu), namely, the x2c-SVPall-s and x2c-TZVPall-s basis sets [79]. The latter were developed on the basis of relativistic Karlsruhe basis sets x2c-XVPall (X = S, TZ) [80] by employing at most four additional functions and optimizing new functions at the scalar exact two-component (X2C) level [81] within the finite nucleus model. The contraction coefficients of the new segments were re-optimized at the X2C level of theory in atomic calculations, resorting to the quasi-Newton algorithm. These basis sets represent a reasonable choice when performing two-component relativistic calculations of NMR shielding constants. However, what choice does one have when performing the 4-component DFT calculations of NMR shielding constants? Using a large, non-specialized basis set optimized for energy at the four-component level in place of a large-component basis set is not an efficient time-preserving way. Another approach that seems reasonable enough is to employ some well-approbated nonrelativistic σ-oriented basis sets in their uncontracted form. This may seem attractive not only for the calculations of the shielding constants per se but also for the relativistic corrections to them. However, what do we know about the applicability of the nonrelativistic, σ-oriented basis sets to such problems? Are the HAHA and HALA relativistic corrections sensitive to the same exponential regions as the nonrelativistic shielding constants? What do we know about their basis set behaviors in general?

A brief mention of the basis set issue for the HALA effect can be found in the work by Malkina et al. [82]. The authors made first attempts to consider the importance of the basis set quality for the description of the spin-orbit HALA (SO-HALA) relativistic corrections to proton shielding constants in hydrogen halides. Malkina et al. suggested that for the correct description of the SO-HALA corrections, it is more important to have a large basis set on hydrogen, as the Fermi-contact (FC) operator probes the spin density at the NMR nucleus, while the basis set on the heavy neighboring atom is of less importance. This is a reasonable suggestion because the leading SO-HALA term contains the molecular integrals of the FC operator in the numerator, like the FC contribution to the nonrelativistic spin-spin coupling constant does. This is a good prerequisite to making the hypothesis that the SO-HALA correction can manifest a similar basis set behavior as the FC term of SSCC in the *s*-angular space.

Overall, this work presents an investigation of the sensitivity of the HALA and HAHA relativistic corrections for shielding constants to changes in different angular spaces of the basis sets set on either heavy or light atoms. Besides, recommendations are given as to what kind of basis sets are better to apply when calculating the NMR shielding constants of light nuclei and the relativistic corrections to them in the systems containing heavy atoms at the four-component DFT level of theory.

## 2. Brief Theoretical Notes

As the relativistic corrections to the NMR shielding constant constitute a subject of our current study, it is pertinent to briefly discuss some notions that will be referred to further in the article.

In the four-component representation, NMR shielding tensors take the form of the polarization propagator in the static limit [34,83]:

(1) $$\sigma_{N;\alpha \beta }=\frac{\mu_{0}e^{2}c^{2}}{4\pi }\langle \langle \frac{{\left({\vec{\alpha }}_{N}\times {\vec{r}}_{N}\right)}_{\alpha }}{r_{N}^{3}};{\left({\vec{\alpha }}_{N}\times {\vec{r}}_{0}\right)}_{\beta }{\rangle \rangle }_{0},$$

where $\vec{\alpha }$ is the Dirac 4×4 matrices, ${\vec{r}}_{N}=\vec{r}-{\vec{R}}_{N}$ and ${\vec{r}}_{0}=\vec{r}-{\vec{R}}_{0}$ represent the radius vectors in relation to the location of the *N*th spectator atom and the origin of the frame of reference, respectively, and the Greek indexes stand for the Cartesian components *x*, *y*, and *z*. Presented in the four-component form like in Equation (1), the $\sigma_{N;\alpha \beta }$ tensor does not allow one to discern common patterns it might follow. Moreover, in the four-component formalism, the physically tractable relativistic corrections of different types cannot be distinguished in an explicit way. For example, a well-known SO-HALA effect on σ-tensor cannot be extracted from Equation (1) without any modifications. In order to pass to a more convenient form, the Hamiltonian of the system is to be converted to the block-diagonal form, resulting in the elimination of the small spinor components [84,85,86,87,88,89]. The alternative way to extract physically meaningful relativistic corrections is to straightforwardly treat the relativistic operators as perturbations operating on the nonrelativistic reference wave function, on the same footing as the magnetic perturbation operators [90,91,92,93].

Briefly, the relativistic corrections to the shielding tensor σ can be divided into two main categories. The first category includes the effects that contribute to the shielding tensor of a light atom due to a neighboring heavy atom, that is, the HALA effect. The second category comprises the relativistic corrections to the shielding tensor of a heavy atom itself, i.e., the HAHA effect [25]. There are typically five contributions to the HAHA effect that are regarded as the leading-order corrections to the shielding constant of a heavy nucleus [90,93,94,95]. These are the so-called scalar corrections, $\sigma^{p-KE/OZ},\;\sigma^{p/mv},\;\;\sigma^{p/Dar}$, and two types of spin-orbit corrections, $\sigma^{FC/OZ/SO}$ and $\sigma^{SD/OZ/SO}$. The three scalar corrections can be thought of as the terms originating from the mixing of the electron spin-independent (or spin-free) PSO (*p*) or PSO-KE (*p*-KE) operator with the spin-free relativistic operators, such as the orbital-Zeeman (*OZ*), mass-velocity (*mv*), and Darwin (*Dar*) terms. At the same time, the two spin-orbit corrections, $\sigma^{FC/OZ/SO}$ and $\sigma^{SD/OZ/SO}$, are the terms originating from the crossing of the hyperfine spin-dependent operators FC and SD with the OZ operator and the spin-dependent spin-orbit (SO) operator. The latter reflects the interactions of the electrons’ spins with the magnetic moments, generated by their orbital motions around the nuclei. The HALA effect also consists of different types of scalar and spin-orbit corrections, though the main part of the spin-orbit HALA (SO-HALA) effect is usually due to the spin-orbit term $\sigma^{FC/OZ/SO}$. Thus, bearing this in mind, we will refer to the $\sigma^{FC/OZ/SO}$ term when reasoning on the SO-HALA effect. The scalar relativistic HALA corrections (we will designate them here as the SF-HALA corrections) to the shielding constants of light atoms were negligible in most cases, especially when the heavy atom belongs to the 16th and 17th groups of the periodic table of elements (PTE) [31].

The physical mechanism that underlies the SO-HALA effect can be described as follows [29,30,96]: in the presence of an external magnetic field, spin-orbit coupling (SOC) on the heavy atom (HA) mixes some triplet character into the closed-shell singlet ground-state wavefunction and thereby spin-polarizes the density distribution in the core and valence shells of the heavy atom, even in a closed-shell molecule. The induced spin density propagates to light (LA) and provides an additional magnetic interaction with a magnetic dipole of LA via the Fermi-contact mechanism, changing the LA NMR chemical shift, σ(LA). In terms of molecular orbitals, the SO-HALA correction can be expressed as follows [29,30,31]:

(2) $$\sigma_{SO-HALA}^{\mu \mu }\left(LA\right)\;\sim \;\sum_{ab}^{vac}\sum_{i}^{occ}\left(\frac{\langle \varphi_{i}|\delta_{LA}|\varphi_{a}\rangle \langle \varphi_{a}|\Delta r_{HA}^{-3}{\hat{l}}_{HA}^{\mu }|\varphi_{b}\rangle \langle \varphi_{b}|{\hat{l}}_{HA}^{\mu }|\varphi_{i}\rangle }{\left(\varepsilon_{i}-\varepsilon_{a}\right)\left(\varepsilon_{i}-\varepsilon_{b}\right)}+perms\right)\;-\sum_{a}^{vac}\sum_{ij}^{occ}\left(\frac{\langle \varphi_{i}|\delta_{LA}|\varphi_{a}\rangle \langle \varphi_{a}|\Delta r_{HA}^{-3}{\hat{l}}_{HA}^{\mu }|\varphi_{j}\rangle \langle \varphi_{j}|{\hat{l}}_{HA}^{\mu }|\varphi_{i}\rangle }{\left(\varepsilon_{i}-\varepsilon_{a}\right)\left(\varepsilon_{j}-\varepsilon_{a}\right)}+perms\right)$$

The denominators in Equation (2) represent the energy gaps of the occupied (*i*, *j*) and vacant (*a*, *b*) molecular orbitals (MOs). The numerators of Equation (2) represent the products of three spatial one-electron molecular integrals, namely, the one-electron spin–orbit (SO) integrals, $\langle \varphi_{a}|\Delta r_{HA}^{-3}{\hat{l}}_{HA}^{\mu }|\varphi_{b}\rangle$, Dirac’s *δ*-function (FC) integrals, $\langle \varphi_{i}|\delta_{LA}|\varphi_{a}\rangle$, and angular momentum or orbital Zeeman (OZ) integrals, $\langle \varphi_{b}|{\hat{l}}_{HA}^{\mu }|\varphi_{i}\rangle$. The subscript “HA” or “LA” refers to a particular operator that is centered on the heavy (HA) or light (LA) atom under consideration, respectively.

Equation (2) is the key expression to make a comparison with the non-relativistic FC term of SSCC that can roughly be presented in the simplest Pople-Santry approximation [97]:

(3) $$J_{FC}\left(HA,LA\right)\sim \sum_{ia}\frac{\langle \varphi_{i}|\delta_{HA}|\varphi_{a}\rangle \langle \varphi_{a}|\delta_{LA}|\varphi_{i}\rangle }{\left(\varepsilon_{i}-\varepsilon_{a}\right)}$$

Equations (2) and (3) contain Dirac’s *δ*-function molecular integrals, $\langle \varphi_{i}|\delta_{LA}|\varphi_{a}\rangle$. At the same time, Dirac’s *δ*-function molecular integrals play a key role in the design of the nonrelativistic *J*-oriented basis sets. Due to these, *J*_FC_(HA,LA) is said to probe the electron density at the positions of NMR active nuclei. That is why a correct description of the FC contribution to SSCC was known to require much higher exponents as compared to those presented in the standard energy-optimized basis sets. By analogy, the same reasoning can be partially applied to the σ_SO-HALA_ as it also contains the matrix elements of the LA*-*centered *δ*-function operator. Thus, one can expect that the basis set behavior of the SO-HALA effect in the *s* region can resemble that of the FC contribution to the corresponding nonrelativistic spin-spin coupling constant between the HA and LA under consideration.

It was also worth mentioning that such a possibility can readily be discerned by applying the simplest restricted kinetic balance (RKB) condition ($\psi_{p}^{S}=\left(1/2c\right)\left(\sigma \hat{p}\psi_{p}^{L}\right)$) [98] to the four-component expression (1). In this case, the characteristic integral in the four-component spinor basis can be expressed as follows:

(4) $$\langle \psi_{p}|\frac{\left[{\vec{\alpha }}_{N}\times {\vec{r}}_{N}\right]}{r_{N}^{3}}|\psi_{q}\rangle =\langle \psi_{p}^{L}|\frac{\left[\vec{\sigma }\times {\vec{r}}_{N}\right]}{r_{N}^{3}}\left(\frac{\vec{\sigma }\hat{p}}{2c}\right)+{\left(\frac{\vec{\sigma }\hat{p}}{2c}\right)}^{+}\frac{\left[\vec{\sigma }\times {\vec{r}}_{N}\right]}{r_{N}^{3}}|\psi_{q}^{L}\rangle \;$$

The central operator of Equation (4) can be thought of as an operator that gives, in particular, the large-component molecular integrals of the Fermi-contact and spin-dipole (SD) operators. This also gives an opportunity to trace a great deal of similarity with the FC term of SSCCs. A similar reasoning can be found in the work by Enevoldsen et al. [99], who compared the operators in the response function for the relativistic 4-component tensor of SSCC with those in the nonrelativistic representation and came to the conclusion that the relativistic interaction operator $\alpha \left[I_{N}\times r\right]/r^{3}$ is responsible for giving matrix elements of the well-known FC+SD operator on the basis of large components. By citing this fact, Enevoldsen et al. explained why they observed almost the same dependence on tight *s*-functions in the relativistic and nonrelativistic calculations of SSCCs, even though the operators look quite different at first sight.

In this paper, we will estimate how far this similarity expands and answer the question: can the existing nonrelativistic *J*-oriented basis sets be recommended for the calculation of the HALA effect in general? We also will show how unsuitable the existing nonrelativistic σ-oriented basis sets may be for the calculation of the HALA effect and consider the effect of their expansion in the tight *s*-region.

## 3. Results and Discussion

### 3.1. General Strategy and Computational Aspects

In order to identify the most suitable basis set type for the four-component calculations of the HALA effect, two important hypotheses have to be proven:

**Hypothesis** **1.** There is a correlation in the behaviors of the HALA effect and the Fermi-contact contribution to the corresponding nonrelativistic one-bond SSCC, ^1^*J*_FC_(HA,LA), upon the saturation of the tight *s*-region in the basis set on the light atom.

**Hypothesis** **2.** *J*-oriented basis sets or σ-oriented basis sets saturated in the tight *s*-region are more suitable for the calculation of the relativistic HALA effect than the existing σ-oriented basis sets or commensurate energy-optimized basis sets.

To reach our goals, this work was carried out in the following order: First of all, we showed the influence of the expansion (saturation) of the basis set on the heavy atom on the relativistic corrections to the shielding constants of light atoms. In particular, by these calculations, we have checked the validity of Malkina’s suggestion [82] that the basis set on the heavy neighboring atom was of less importance for the HALA effect. At the same time, we monitored the behavior of the relativistic correction to the heavy atom shielding constants (HAHA effect).

Once the saturation of the basis set on the heavy atom was completed, we set the final basis set on the heavy atom and proceeded to the next stage, where we carried out the saturation of the basis set used on the light atoms. In that stage, we showed what exponential regions mostly affect the HALA effect and gave the answer to the question: is there a correlation between the behavior of the nonrelativistic FC contribution to the spin-spin coupling constant *J*_HA,LA_ and that of the HALA effect, σ_HALA_, in the *s*-angular space?

In the final stage, we have tested different basis sets on their suitability for the calculation of the HALA effect on proton and carbon shielding constants at the 4c-DFT level of theory and checked whether the conclusions made for the HALA effect can also be applied to the shielding constants of the light atoms.

In the basis set saturation procedures, we used the following molecules: HI (**1**), I-CH_3_ (**2**), SbH_2_-CH_3_ (**3**), InH_2_-CH_3_ (**4**), SnH_3_-CH_3_ (**5**), and TeH-CH_3_ (**6**) as model compounds. In the rest of the calculations, we used a wider series of molecules, consisting of compounds **1**–**6** and five aromatic compounds: C_6_H_5_I (**7**), C_5_H_5_Sb (**8**), C_5_H_6_Sn (**9**), C_6_H_5_InH_2_ (**10**), and C_4_H_4_Te (**11**).

All shielding calculations were performed using the SVWN5 exchange-correlation functional [100,101]. This represents the local density approximation (LDA) [102,103], in which the exchange was uniquely defined analytically in the form of the exchange energy of a homogeneous electron gas while its correlation term was defined through several parameterizations, mostly relying on the highly accurate quantum Monte-Carlo simulations [104]. We have chosen this function based on two prerequisites. The strongest argument was based on an observation that we made in one of our previous papers [105]. Namely, it was shown that the SVWN5 functional provides the best description of the relativistic effects on tellurium NMR chemical shifts, in spite of the fact that it has been designed neither for the relativistic effects nor for the NMR shielding constants. On the other hand, we are also aware of the work by Lutnæs et al. [106], who showed that the LDA model appears to be particularly stable towards triplet instabilities [107], reflecting a balanced description of exchange and correlation. The latter is very important for the calculation of triplet excitation properties such as the FC and SD contributions to the spin-spin coupling constants or the SO-HALA effect.

The relativistic corrections to shielding constants were evaluated as the differences between the four-component relativistic DFT values and those obtained within the nonrelativistic scheme. For the nonrelativistic regime, the “10c limit scheme” was employed, which implies the increasing of the speed of light by 10 times in the relativistic four-component calculations. The gauge-including atomic arbitals (GIAO) approach [108,109] has been applied to circumvent the problem of the dependence of shielding constants on the gauge origin [110,111]. In the full four-component calculations, the unrestricted kinetic balance (UKB) [112,113] condition was used to generate the small component basis set. This was done in order to recover the magnetic balance condition [114,115,116] as far as possible, in line with the findings of Olejniczak et al. [117], who proved that the UKB recovers to a high extent the magnetic balance condition [115,116,118] if it is applied in combination with the GIAO formalism.

In the first two stages, Dyall’s relativistic basis set of double-zeta quality, dyall.v2z [119,120], has been chosen as the starting basis set to carry out our numerical experiments on the expansion of angular spaces. The dyall.v2z basis set was optimized in the Dirac-Hartree-Fock calculations by minimizing the degeneracy-weighted average of the energies of the atomic states arising from the principal nonrelativistic valence electron configuration using the Gaussian nuclear charge distribution model [121], followed by the MR-SDCI [122,123] optimizations of the valence correlating functions. The configuration of the original dyall.v2z basis set for hydrogen, carbon, and 5*p* elements is as follows: [6*s*1*p*], [10*s*6*p*1*d*], [21*s*15*p*11*d*], respectively.

An approach to which we were resorting here implies a consecutive augmentation of each angular space of the dyall.v2z basis set with additional tight and diffuse functions. We have chosen the double-zeta quality basis set for the saturation procedure because the triple- or higher-level basis sets were already large enough in all angular spaces to provide a great deal of completeness in the description of the relativistic corrections, thus hampering the picture of the possible sensitivity of these to the presence or absence of the basis set functions of various types. Only if the convergence of the relativistic correction under study is achieved within a certain angular space do we pass to the next space, keeping previously saturated spaces fixed. The exponent of the additional *i-*th tight or diffuse function *ζ_i_* was evaluated in accordance with the geometric progression or even-tempered series [124], *ζ_i_* = *αβ^i^* or *ζ_i_* = *α/β^i^*, respectively. Accordingly, in these formulae, *α* represents the largest or smallest exponent *ζ_n_* in the original basis set, and *β* is the ratio of the two largest or two smallest exponents, *β* = *ζ_n_*/*ζ*_*n*−1_ or *β* = *ζ*_*n*−1_/*ζ_n_*, for the tight or diffuse regions, respectively. This produces an exponential sequence with a fixed ratio between two adjacent exponents and the guarantee that no two Gaussians have excessive overlap, in line with the suggestions of Reeves and Fletcher [124].

### 3.2. Changes in the HALA and HAHA Effects upon the Expansion of the Basis Set on the Heavy Atom

In this section, we investigate how the expansion of the basis set on the heavy atom affects the relativistic corrections to the shielding constant of the neighboring light atoms (HALA effect). In this way, we discuss here only the results obtained for the one-bond HALA or *α*-HALA effect, though the preliminary studies for the multiple-bond or distant HALA effects have also been carried out. In the preliminary studies, we have found out that the changes in the distant HALA effects upon expanding the basis set on heavy atoms were so small that they were not worth attention.

**Figure 1:**
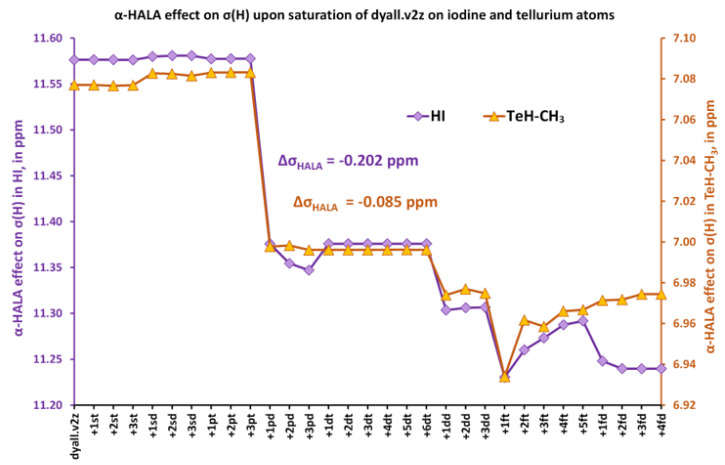
The change of the α-HALA effect on the proton shielding constants of HI (left vertical axis) and TeH-CH_3_ (right vertical axis) upon saturation of the dyall.v2z basis set are used on the iodine and tellurium atoms, respectively.

**Figure 2:**
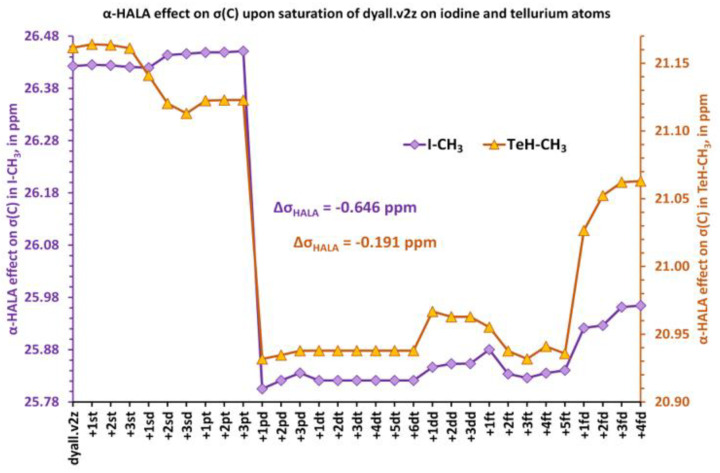
The change of the α-HALA effect on the carbon shielding constants of I-CH_3_ and TeH-CH_3_ upon saturation of the dyall.v2z basis set is used on the iodine and tellurium atoms, respectively.

We considered six representatives: HI (**1**), I-CH_3_ (**2**), SbH_2_-CH_3_ (**3**), InH_2_-CH_3_ (**4**), SnH_3_-CH_3_ (**5**), and TeH-CH_3_ (**6**). Initially, we set the dyall.v2z basis set on all atoms. Then, we fixed the basis set on the hydrogen and carbon atoms and started to vary the basis set on the heavy atom. The effects of the consecutive saturation of each angular space of the basis set on the heavy atom on the *α*-HALA correction to the proton and carbon shielding constants of HI and TeH-CH_3_ and I-CH_3_ and TeH-CH_3_, respectively, are illustrated in Figure 1 and Figure 2. The data for the rest of the model compounds (**3**, **4**, and **5**) are given in the supporting information file (see Tables S2 and S3).

The notation “+ *nk*l”, that was used in all figures in this work, means adding *n* functions of *k*-type (*k* = *s*, *p*, *d*, …) to the previous basis set, with *l* designating a particular region within the angular space, namely, *l* = “t” or “d” for t = “tight” and d = “diffuse”, respectively. From these figures, it follows that the expansion of the diffuse *p* region causes the most pronounced influence on the HALA effect on the ^1^H and ^13^C NMR shielding constants. The most noticeable effect can be observed for two iodine molecules, HI and I-CH_3_. In the first case, the proton α-HALA effect in HI drops by 0.202 ppm upon adding only one diffuse *p*-function, while the decrease of the carbon α-HALA effect in I-CH_3_ was about 0.646 ppm. We also artificially added a new *f*-region that had not been presented in the original dyall.v2z basis set for heavy elements to check if there would be any difference. These functions can be considered the polarization functions of the original basis set. The initial starting exponents were taken from dyall.v3z [125] and the ratio used to generate new exponents in the *f*-region was taken from the dyall.v4z basis set [126]. One can notice that the effect of adding *f*-functions was negligible at most, but could be compared to the effect of the additional diffuse *p*-functions in the case of the carbon α-HALA effect in TeH-CH_3_. For the rest of the model compounds (**3**, **4**, and **5**), the changes in proton and carbon *α*-HALA effects were chaotic and negligible, varying within the range of 0.03 and 0.27 ppm in the former and latter cases, respectively. In general, the influence of the basis set used on the heavy atom on the relativistic correction to the shielding constant of the neighboring light atom was insignificant. In this respect, this conclusion corroborates the observation of Malkina et al. [82], who claimed that the basis set on the heavy neighboring atom was of less importance for the description of the HALA effect. Only in the case of the most powerful source of the HALA effect among the 5*p*-block elements, namely, the iodine atom, was the diffuse *p*-region of the dyall.v2z basis set worth expanding by only one function in order to totally recover the deficiency of the diffuse *p*-functions.

**Figure 3:**
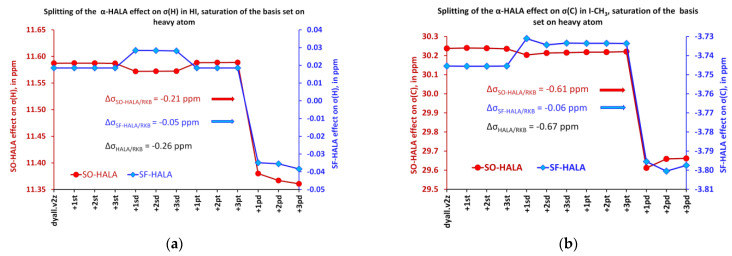
Splitting of the α-HALA effect into SO and SF contributions while saturating the heavy basis set in: (**a**) HI; (**b**) I-CH_3_.

To get a deeper insight on the origin of the change in the diffuse *p*-region, we have split the HALA effect into two contributions, SO-HALA and SF-HALA, and monitored the behavior of both upon a sequential saturation of the basis set on the iodine atom in HI and I-CH_3_ molecules. For that purpose, we have calculated both the SF-HALA and full HALA effects at the four-component DFT-SVWN5 level, using, though, the restricted kinetic balance (RKB) condition. The SO-HALA effect was evaluated as the difference between the full HALA and SF-HALA corrections. The values of the full HALA effect calculated with the RKB condition appeared to be only slightly different from those obtained with the UKB condition; hence, in our opinion, using the RKB in the splitting procedure should hardly cause any bias in the qualitative considerations. It was found that in both cases the SO-HALA contribution overwhelmingly predominates over the SF-HALA term and that the noticeable drop in the diffuse *p*-region was due to the simultaneous decrease of both SO-HALA and SF-HALA counterparts, which provide ca. 80% and 20% of the total decrease, respectively (see Figure 3).

Presumably, the additional diffuse *p*-type function improves the description of two iodine *π*-type lone pairs that are known to play a decisive role in the SO-HALA effect, while the contributions from the *σ*-type lone pair were shown to be very small [23,127].

**Figure 4:**
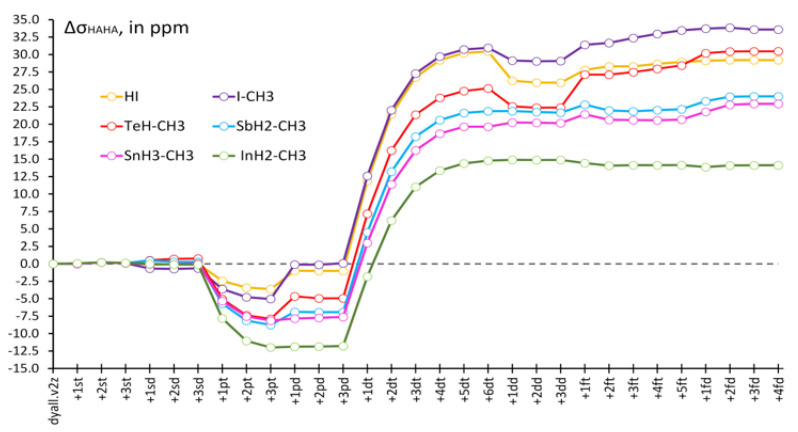
Relative changes of the HAHA effects in model compounds **1–6** upon saturation of the basis set on the heavy atom with respect to the corresponding starting values, calculated using the unsaturated dyall.v2z basis set.

Another important observation made by us while saturating the heavy basis set consists of a drastic increase of the HAHA effect on the shielding constants of all considered heavy nuclei in the tight *d*-region. It was difficult to illustrate the basis set behaviors of the HAHA effects for all nuclei in one plot due to a significant difference in their scales; therefore, for each molecule, we have evaluated the deviations (Δσ_HAHA_) of the values of the HAHA effect at each point from those calculated at the starting point (corresponding to the original dyall.v2z basis set) and plotted them in Figure 4. Thus, one can see that the relative changes of the HAHA effects in six model compounds follow the same pattern upon saturation of the basis set on the heavy atom.

One can notice that there is a decrease of the HAHA effect in the tight *p*-region, with varying magnitudes, inherent to each type of heavy atom. The decrease of the HAHA effect in the tight *p*-region can be said to follow the atomic number trend. The most pronounced decrease was observed for the indium compound (Z = 49), followed by somewhat lesser and almost equal decreases for Sn (Z = 50) and Sb (Z = 51), then comes Te (Z = 52), and, finally, the least decrease of the HAHA effect was observed for the iodine (Z = 53) compounds. The saturation of the tight *d*-region, on the contrary, resulted in a significant increase in the HAHA effects on all nuclei. At that point, the most pronounced increase was observed for the iodine compounds (by about 30 ppm), and the least pronounced increase was observed for the indium compound (by about 25 ppm). In order to put these figures into the absolute scale of the HAHA effect, it is pertinent to mention here the exact values of the HAHA effect calculated in the starting points (with unsaturated dyall.v2z basis set) for different nuclei in compounds **1**–**6**. Namely, these are as follows: 1226 ppm for σ(I) in HI, 1242 ppm for σ(I) in I-CH_3_, 1001 ppm for σ(Te) in TeH-CH_3_, 816 ppm for σ(Sb) in SbH_2_-CH_3_, 756 ppm for σ(Sn) in SnH_3_-CH_3_, and 645 ppm for σ(In) in InH_2_-CH_3_. Thus, the changes of about 25–30 ppm of the HAHA effect on different nuclei occurring during the saturation of the tight *d*-region represent ca. 2–4% of the total values of the HAHA effect calculated at the starting point. These changes can hardly be considered negligible. Based on these studies, we conclude that it is of paramount importance to take special care of the tight *d*-region and, to some extent, of the tight *p*-region in the future developments of the specialized basis sets of double-zeta quality for the calculations of relativistic effects on the shielding constants of heavy nuclei. In this respect, the dyall.v3z basis set [125] can be regarded as the basis that is complete enough in all areas and can be used for the high-quality calculations of the HAHA effect.

**Figure 5:**
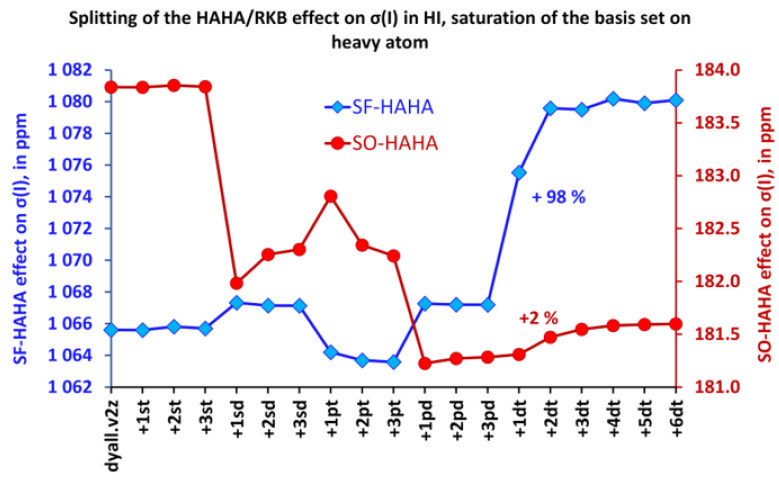
Splitting of the HAHA effect into SO and SF contributions in the HI molecule while saturating the basis set on the iodine atom.

As opposed to the HALA effect, the HAHA effect was determined mostly by the scalar contributions, and its increase in the tight *d*-region was due to the drastic growth of the SF-HAHA contribution in all cases. This is illustrated in Figure 5, which shows the behaviors of the SO- and SF-HALA effects on the iodine shielding constant of the HI molecule upon saturation of the basis set on the iodine atom.

The saturated configurations for the basis sets on heavy atoms, deduced based on the convergence of both types of relativistic effects (HALA and HAHA) in each angular space (without taking into account insignificant alternations in the *f*-region), are compiled in Table 1. These are used in the next part of this work.

### 3.3. Changes in the HALA Effect upon the Expansion of the Basis Set on the Light Atom

**Figure 6:**
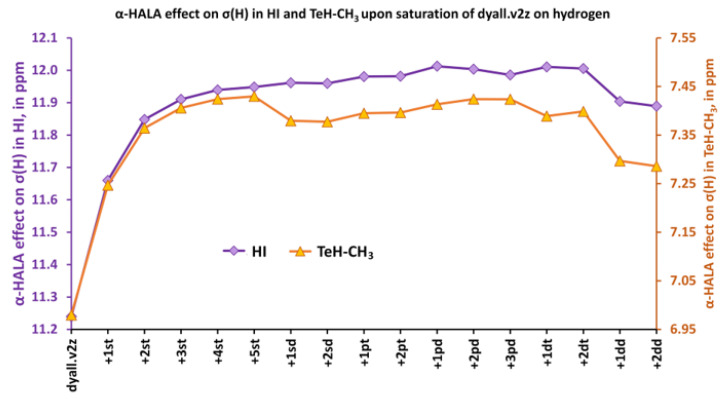
The variation of the α-HALA effect on proton shielding constant upon saturation of the dyall.v2z basis set on hydrogen atoms in molecules **1** and **6**.

**Figure 7:**
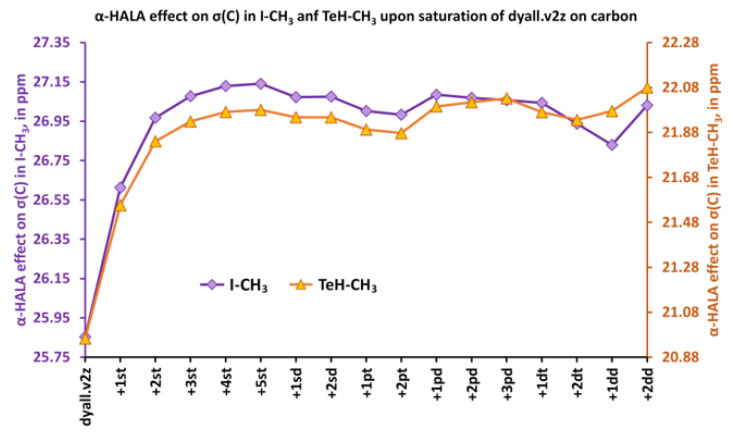
The variation of the α-HALA effect on the carbon shielding constant upon saturation of the dyall.v2z basis set on the carbon atom in molecules **2** and **6**.

In this section, we have fixed the saturated basis sets on the heavy atoms in six model compounds and expanded the basis set on the light atoms. The saturations of the basis sets on the hydrogen and carbon atoms were carried out separately. This implies setting the dyall.v2z basis set on hydrogens when saturating carbon basis set and vice versa. In all cases, we observed a significant sensitivity of the α-HALA effects to the expansion of the tight *s*-region. This is exemplified here by the changes in the α-HALA effect on proton and carbon shielding constants upon saturation of the dyall.v2z basis set on the atoms in question in molecules **1**, **6** and **2**, **6**, respectively (see Figure 6 and Figure 7). The data for the rest of the model compounds are compiled in the supporting information file (see Tables S4 and S5).

Evidently, only the tight *s*-region may be of interest here; the alternation of the rest of the functional space does not affect the α-HALA effect in the least. As was expected, the sensitivity of α-HALA effect to the expansion of the tight *s*-region turned out to be completely determined by the SO-HALA contribution in all cases. The results of splitting the α-HALA effect on proton and carbon shielding constants of HI (**1**) and I-CH_3_ (**2**) into SO-HALA and SF-HALA contributions are collected in the supporting information file; see Figures S6 and S7.

**Figure 8:**
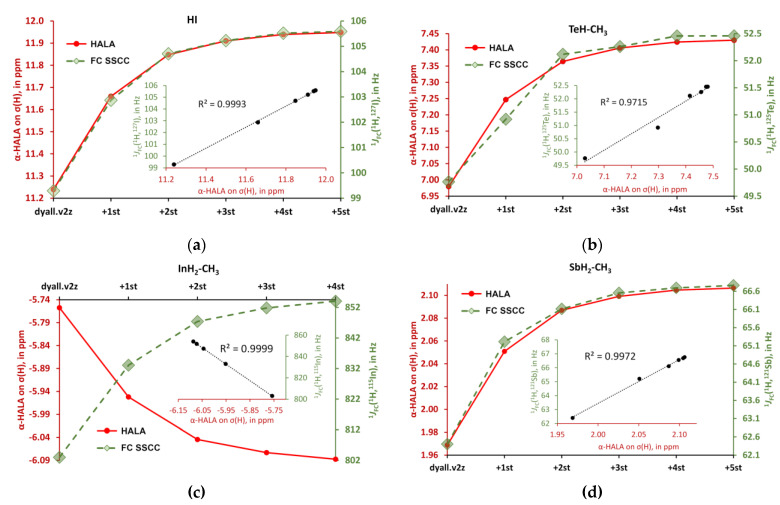
The behaviors of the α-HALA effects on σ(^1^H) upon the saturation of the dyall.v2z basis set on protons in the tight *s*-region against that of the ^1^*J*_FC_(HA,^1^H) in molecules: (**a**) HI (**1**); (**b**) TeH-CH_3_ (**6**); (**c**) InH_2_-CH_3_ (**4**); and (**d**) SbH_2_-CH_3_ (**3**).

**Figure 9:**
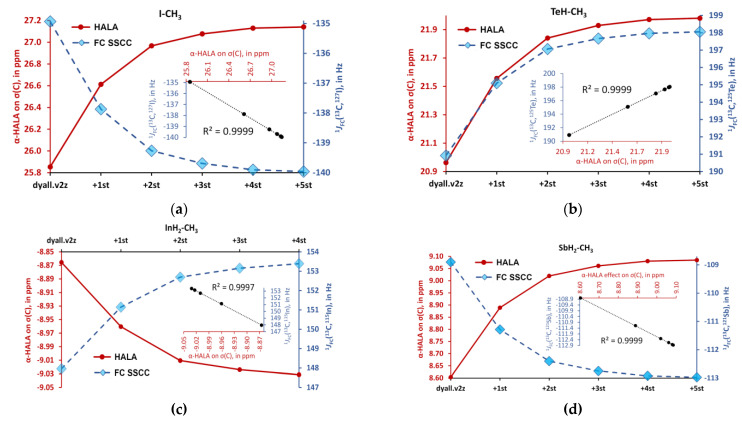
The behaviors of the α-HALA effects on σ(^13^C) upon the saturation of the dyall.v2z basis set on carbons in the tight *s*-region against that of the ^1^*J*_FC_(HA,^1^H) in molecules are: (**a**) I-CH_3_ (**2**); (**b**) TeH-CH_3_ (**6**); (**c**) InH_2_-CH_3_ (**4**); and (**d**) SbH_2_-CH_3_ (**3**).

The observed sensitivity signifies that our **Hypothesis 1** could be right, and one could expect a great deal of similarity in the light basis set behaviors of the α-HALA effect and the Fermi-contact contribution to the corresponding nonrelativistic SSCC in the tight *s*-region. To prove that, we have plotted these behaviors in the cojoined diagrams, with the left axis corresponding to the α-HALA effect on σ(^1^H) or σ(^13^C) and the right axis corresponding to the ^1^*J*_FC_(HA,LA), where HA = ^115^In, ^119^Sn, ^121^Sb, ^125^Te, ^127^I, and LA = ^1^H or ^13^C. Figure 8 and Figure 9 illustrate the behaviors of the α-HALA effect on σ(^1^H) or σ(^13^C) upon the saturation of the dyall.v2z basis set on protons or carbons in the tight *s*-region against that of the ^1^*J*_FC_(HA,^1^H) or ^1^*J*_FC_(HA,^13^C) in compounds **1**, **3**, **4**, **6** and **2**, **3**, **4**, **6**, respectively. The calculations of SSCCs have been carried out at the nonrelativistic DFT-SVWN5 level of theory, using the same basis set schemes as in the shielding calculations. The rest of the data was less representative due to the smallness of the HALA effects.

Overall, from Figure 8 and Figure 9, one can see that there is a definite correlation between the two quantities under study when saturating the tight *s*-region of the basis set on a light atom. The quadratic Pearson correlation coefficient, R^2^, was above 0.999 in practically all cases (with two exceptions for the cases depicted in Figure 8b,d, where R^2^ = 0.9715 and 0.9972, respectively). These results confirm **Hypothesis 1**. As a direct consequence of this, one can expect a great deal of similarity between the general trends of the HALA effect on σ(LA) and the *J*_FC_(HA,LA) on the variety of basis sets used on the light atoms. Indeed, the FC contributions to SSCCs are known to be very sensitive to the basis set used on the coupling nuclei [54,128,129,130]. In particular, they are described much better with the specialized *J*-oriented basis sets (that are usually reinforced in the tight *s*-region) as compared to small, nonspecialized basis sets. The difference between the values of *J*_FC_(HA,LA) evaluated with different basis sets can be very substantial. Given the almost 100% correlation between the behaviors of the α-HALA effect and the corresponding ^1^*J*_FC_(HA,LA) that we have observed in six model compounds upon saturating the tight *s*-region, we can now assume that there can also be a great deal of coherence in the sensitivity of these two quantities towards the type of basis set used on light atoms.

To check this out, we extended our series of six model compounds (**1**–**6**) with five aromatic compounds: C_6_H_5_I (**7**), C_5_H_5_Sb (**8**), C_5_H_6_Sn (**9**), C_6_H_5_InH_2_ (**10**), and C_4_H_4_Te (**11**), and calculated the α-HALA effect on the shielding constants of hydrogen and carbon atoms directly bonded to the heavy atom together with the FC contributions to the corresponding one-bond SSCCs in the whole series. Distant HALA effects and the corresponding multiple-bond SSCCs have also been calculated; however, we did not discuss them here due to the insignificance of the former in the majority of cases.

In these calculations, we altered the basis set on the light atoms while keeping the same basis set on the heavy atom unchanged. Namely, the dyall.av3z basis set [125] was used on the heavy atom, while a variety of twenty different basis sets was used on the light atoms. We considered the dyall.av3z basis set to be sufficient for the description of the heavy atoms in the calculation of the HALA effect because, for the 5*p*-elements, it has the composition [29*s*22*p*16*d*2*f*], and this overwhelmingly replenishes the need of the functions in all angular spaces, as in accordance with our current studies on saturation.

All considered basis sets for light atoms are listed in Table 2, including their types, their configurations for hydrogen and carbon atoms, and their total sizes (*N*_bas_) for both atoms. The considered basis sets are split into four types. Type ***A*** stands for the specialized *J*-oriented basis sets, type ***B****—*for specialized σ-oriented basis sets, type ***C****—*for the specialized σ-oriented basis sets profoundly extended in the tight *s*-region, and type ***D*** comprises nonspecialized, energy-optimized basis sets. All basis sets were used in the uncontracted form in both the relativistic HALA and nonrelativistic SSCC calculations. This was done in order to avoid the incoherence of the study due to the basis-set contraction error in the case of nonrelativistic calculations.

**Figure 10:**
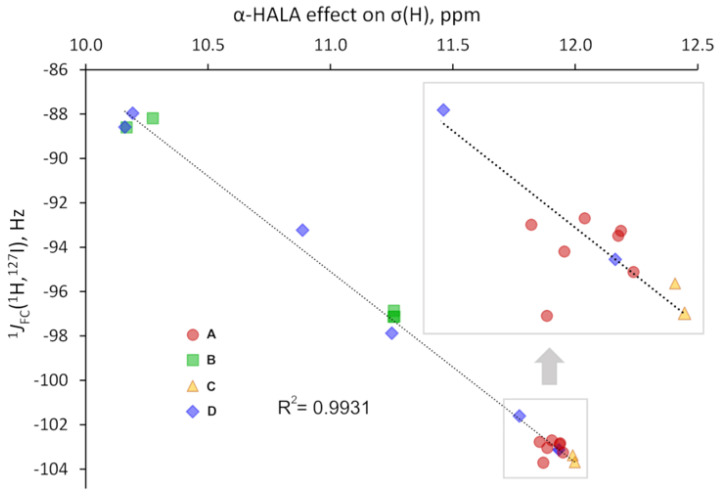
The correlation plot for the α-HALA effect from the iodine atom on the σ(^1^H) and the corresponding ^1^*J*_FC_(^1^H,^127^I) in HI upon varying the basis set on the light hydrogen atom.

**Figure 11:**
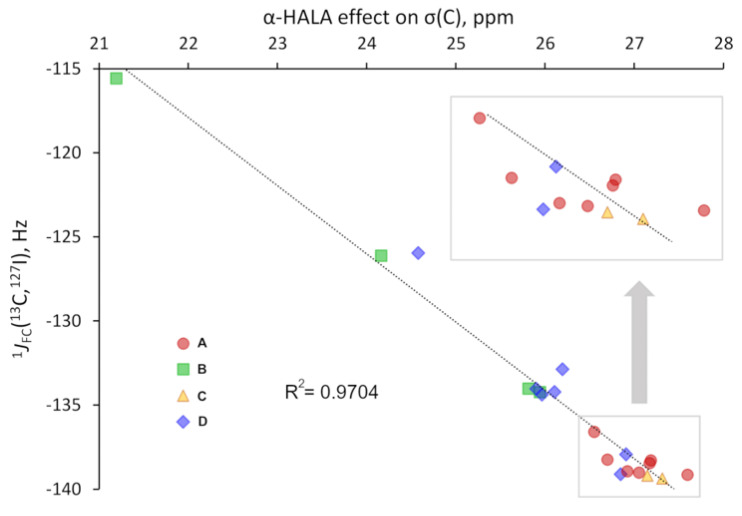
The correlation plot for the α-HALA effect from the iodine atom on the σ(^13^C) and the corresponding ^1^*J*_FC_(^13^C,^127^I) in I-CH_3_ upon varying the basis set on the carbon atom.

All results are collected in Tables S8–S23 in the Supporting Information file, while here we present only the correlation plots for the values of the most pronounced α-HALA effect from the iodine atom on the proton and carbon shielding constants in HI and I-CH_3_, respectively, and the corresponding ^1^*J*_FC_(LA,^127^I) upon varying the basis set on LAs, see Figure 10 and Figure 11.

As one can see from Figure 10 and Figure 11, there is a strong correlation in both cases, namely between the α-HALA effect on σ(^1^H) and ^1^*J*_FC_(^1^H,^127^I) in HI (R^2^ = 0.9931) and between the α-HALA effect on σ(^13^C) and ^1^*J*_FC_(^13^C,^127^I) in I-CH_3_ (R^2^ = 0.9704). Even more so, we see that the points corresponding to the basis sets of ***A*** and ***C*** types (red circles and yellow triangles, respectively) were clearly located apart from those corresponding to the basis sets of ***B*** and ***D*** types (green squares and blue diamonds, respectively)**.** The only two exceptions in both cases were the points corresponding to the largest ***D***-type basis sets, namely, dyall.aae4z and dyall.v3z, which were so large in size that they can be regarded as providing the most reliable data in the sense of basis set issue. In this respect, it is very appropriate that the points corresponding to the specialized *J*-oriented basis sets (***A***-type) and the σ-oriented basis sets with extended tight *s*-region (***C***-type) were in the same areas in plots 10 and 11, with the points corresponding to the best dyall.aae4z basis set.

It was also interesting to note that there was a significant difference between the values of the HALA effect obtained with different types of basis sets. For instance, as can be seen from plots 10 and 11, these differences reach up to ca. 2 and 7 ppm for the α-HALA effect on the proton and carbon shielding constants of HI and I-CH_3_, respectively. This once again speaks in favor of the utmost importance of the correct choice of the basis set for the appropriate calculation of the HALA effect.

A high degree of correlation between the HALA effect and the corresponding FC SSCC in the sense of the basis set used on the light atom signifies that using the basis sets of ***A*** and ***C*** types could represent the best choice not only for the calculation of the nonrelativistic FC SSCCs but also for the HALA effect.

In general, the results obtained in this section corroborate **Hypothesis 1**. Bearing this in mind, we pass to the final step, where we will prove the **Hypothesis 2**, which can be thought of as a direct consequence of the observations made in this section.

### 3.4. Suitability of Different Types of Basis Sets to the Calculation of the HALA Effect and Shielding Constants of Light Nuclei

Our goal in this section was to prove **Hypothesis 2**, which consists in the statement: “*J*-oriented basis sets or σ-oriented basis sets saturated in the tight *s*-region were more suitable for the calculation of the relativistic HALA effect than the existing σ-oriented basis sets or commensurate energy-optimized basis sets.” To achieve the goal, we calculated the HALA effect for all light atoms in the series **1**–**11** (with averaging the values for the labile groups) using the basis sets **2**–**20** (see Table 2) on light atoms and dyall.av3z on the heavy atom. The obtained values were compared with the reference values, which have been evaluated using the quadruple-zeta quality dyall.aae4z basis set on all atoms. As was already mentioned, the dyall.aae4z basis set can be regarded as the best one in the shielding constant calculations because all the angular momentum shells of this basis were already complete enough to provide the converged values close to the CBS limit. Its configurations for the 1*s*, 2*p*, and 5*p*-elements are as follows: [12*s*4*p*3*d*2*f*], [19*s*11*p*6*d*4*f*2*g*], and [34*s*28*p*19*d*12*f*7*g*2*h*], respectively. The average deviations of the HALA effects on proton and carbon shielding constants calculated with different basis sets from the corresponding reference values were estimated as MAEs, or mean absolute errors. The similar MAEs were also evaluated for all proton and carbon shielding constants of molecules **1**–**11**.

**Figure 12:**
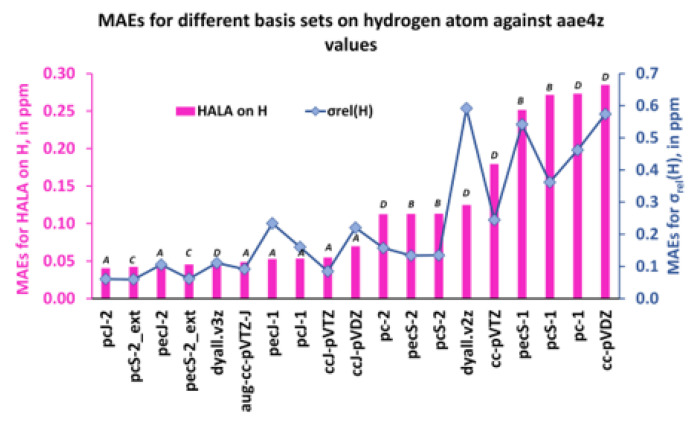
The MAEs for the HALA effect on the proton shielding constants and that of the proton shielding constants of molecules **1**–**11** were calculated with different basis sets on light atoms against the values obtained with the dyall.aae4z basis set on all atoms.

**Figure 13:**
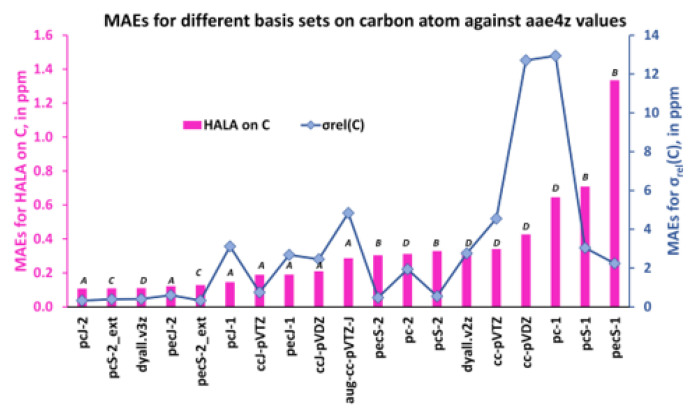
The MAEs for the HALA effect on the carbon shielding constants and that of the carbon shielding constants of molecules **1**–**11** were calculated with different basis sets on light atoms against the values obtained with the dyall.aae4z basis set on all atoms.

Thus, the MAEs for the HALA effect and for the relativistic shielding constants of the hydrogen and carbon nuclei are shown in Figure 12 and Figure 13 in ascending order.

As one can see from Figure 12 and Figure 13, the first five least MAEs for both the HALA effect and the shielding constants of carbon and hydrogen nuclei are provided by the same group of basis sets of ***A*** and ***C*** types, though, including one basis set of ***D*** type. These basis sets are as follows: pcJ-2 (***A***), pecJ-2 (***A***), pcS-2_ext (***C***), pecS-2_ext (***C***), and dyall.v3z (***D***). The common peculiarity of these basis sets consists in a large number of *s*-type functions, with a considerable tightness in the largest of them. The dyall.v3z basis set was also on par with the best basis sets of ***A*** and ***C*** types because it was one of the largest basis sets among the considered ones and provided enough flexibility in all angular spaces, including the most important tight *s*-region. The next five basis sets that provided the results of slightly less accuracy for the HALA effect in relation to the reference values were all of the ***A***-type in both cases. The rest of the considered basis sets were all of the ***B*** or ***D*** types, with four double-zeta quality basis sets, cc-pVDZ (***D***-type), pc-1 (***D***-type), pcS-1 (***B***-type), and pecS-1 (***B***-type), providing the largest MAEs.

Also, as one can see from Figure 12 and Figure 13, general MAE trends for the proton and carbon shielding constants follow the MAE trends for the corresponding HALA effects, being somewhat different only in particular cases. The same five basis sets, pcJ-2, pecJ-2, pcS-2_ext, pecS-2_ext, and dyall.v3z, provide the best accuracy, but starting with the sixth basis set from the left, there were some discrepancies. In particular, the basis sets of triple-zeta quality, namely, ccJ-pVTZ (***A***-type), pcS-2 (***B***-type), and pecS-2 (***B***-type), provide almost the same accuracy as the five best ones, while the double-zeta quality basis sets of ***B***-type, pcS-1, and pecS-1 are not among the poorest basis sets for the description of the carbon shielding constants (their accuracy is comparable with that of the pecJ-1 or ccJ-pVDZ basis sets). Be as it may, the common observation made for the HALA effect does not fail in the case of the relativistic shielding constants of light nuclei: in general, the best results were provided by the *J*-oriented basis sets.

These results confirm **Hypothesis 2**, and we conclude here that the best description of both the HALA effect and the corresponding shielding constant of LA at the four-component DFT level was achieved with either the *J*-oriented basis sets (***A*** type) or the σ-oriented basis sets saturated in the tight *s*-region (***C*** type). At that, the basis sets of triple-zeta quality, pcJ-2, pecJ-2, pcS-2_ext, and pecS-2_ext, were the best among the best basis sets of ***A*** and ***C*** types for both quantities. The dyall.v3z basis set was also among the best, but this was due to its large size and high flexibility.

As the simplest approach, we recommend using the existing *J*-oriented basis sets for the four-component relativistic calculations of the shielding constants of light nuclei as well as for the calculations of the relativistic HALA corrections to them if there are heavy atoms in the system.

## 4. Materials and Methods

All geometry optimizations were performed at the relativistic four-component DFT-PBE0 [133,134]/dyall.av3z level without taking into account media effects (gas phase), within the DIRAC program [135]. All equilibrium geometries are presented in the supporting information file (see Table S1).

All calculations of NMR shielding constants were carried out at the four-component GIAO-DFT(SVWN5) level of theory in the DIRAC program. All calculations of SSCCs were carried out at the nonrelativistic DFT(SVWN5) level of theory in the Dalton program [136].

## 5. Conclusions

On the example of 5*p*-block elements considered heavy atoms and hydrogen and carbon atoms considered light ones, the HALA effect was shown to be practically insensitive to the basis set on the heavy atom. Only in the case of the most powerful source of the HALA effect among the 5*p*-block elements, namely, the iodine atom, have we noticed a small change (of about −0.2 and −0.6 ppm for the hydrogen and carbon nuclei, respectively) of the HALA effect upon expanding the dyall.v2z basis set on the iodine with one diffuse *p*-function. In the meantime, a strong sensitivity of the HALA effect to the expansion of the tight *s*-region of the basis set used on the light atom has been observed. It was shown that there is a definite correlation between the behaviors of the HALA effect and the Fermi-contact contribution to the corresponding nonrelativistic one-bond SSCC, ^1^*J*_FC_(HA,LA), when varying the tight *s*-region of the basis set used on the light atom. Thus, the *J*-oriented basis sets as well as the σ-oriented basis sets saturated in the tight *s*-region were shown to be more suitable for the calculation of the relativistic HALA effect than the existing σ-oriented basis sets or commensurate energy-optimized basis sets. The triple-zeta quality basis sets of these types, namely, pcJ-2, pecJ-2, pcS-2_ext, and pecS-2_ext, were found to perform the best for both the shielding constants of light nuclei and the relativistic HALA corrections to them. As the simplest approach, the existing *J*-oriented basis sets were recommended for the four-component relativistic calculations of both quantities.

The relativistic HAHA effect was found to be hardly sensitive to the *s*-region of the basis set on the heavy atom. At the same time, tight *d*-region and, to a lesser extent, tight *p*-region were found to be insufficient in the dyall.v2z basis set for heavy 5*p*-block elements to properly describe the relativistic effects on the shielding constants of the corresponding heavy nuclei. Therefore, one should take special care of the tight regions of the *p*- and, especially, of the *d*-angular spaces in the future developments of the specialized basis sets of double-zeta quality for the shielding constants of heavy nuclei. In the current state of affairs, the dyall.v3z basis set can be recommended for the HAHA calculations because it is already complete enough in all angular spaces.

## Figures and Tables

**Table 1:** Configurations of saturated heavy basis sets ^1^.

Atom	Modifications	Saturated Configuration
I	+ 3*s*d + 2*p*t + 2*p*d + 5*d*t + 2*d*d	[24*s*19*p*18*d*]
Te	+ 1*s*t + 2*s*d + 3*p*t + 3*p*d + 5*d*t + 2*d*d	[24*s*21*p*18*d*]
Sb	+ 3*s*d + 2*p*t + 1*p*d + 6*d*t + 1*d*d	[24*s*18*p*18*d*]
Sn	+ 3*p*t + 1*p*d + 5*d*t + 2*d*d	[21*s*19*p*18*d*]
In	+ 1*s*t + 1*s*d + 3*p*t + 6*d*t + 1*d*d	[23*s*18*p*18*d*]

^1^ The original dyall.v2z basis set for 5*p*-elements is of the following configuration: [21*s*15*p*11*d*].

**Table 2:** Basis sets used for testing. Types of basis sets: ***A***—specialized *J*-oriented; ***B***—specialized σ-oriented; ***C***—σ-oriented extended in a tight *s*-region; ***D***—nonspecialized, energy-optimized.

#	Basis Set	Type	Configuration for H, *N*_bas_	Configuration for C, *N*_bas_	Ref.
1	dyall.aae4z	***D***	[12*s*4*p*3*d*2*f*]	[19*s*11*p*6*d*4*f*2*g*]	[120]
2	aug-cc-pVTZ-J	***A***	[10*s*3*p*1*d*], 24	[15*s*6*p*3*d*1*f*], 55	[53,69]
3	ccJ-pVDZ	***A***	[6*s*2*p*], 12	[11*s*5*p*2*d*], 36	[61]
4	ccJ-pVTZ	***A***	[7*s*3*p*2*d*], 26	[12*s*6*p*3*d*1*f*], 52	[61]
5	pcJ-1	***A***	[6*s*2*p*], 12	[9*s*5*p*2*d*], 34	[59]
6	pcJ-2	***A***	[8*s*3*p*2*d*], 27	[12*s*7*p*3*d*2*f*], 62	[59]
7	pecJ-1	***A***	[7*s*2*p*], 13	[10*s*5*p*2*d*], 35	[65]
8	pecJ-2	***A***	[8*s*3*p*1*d*], 22	[11*s*6*p*3*d*1*f*], 51	[65]
9	pcS-1	***B***	[4*s*2*p*], 10	[7*s*5*p*1*d*], 27	[51]
10	pcS-2	***B***	[6*s*3*p*1*d*], 20	[10*s*7*p*2*d*1*f*], 48	[51]
11	pecS-1	***B***	[4*s*2*p*], 10	[7*s*5*p*1*d*], 27	[75]
12	pecS-2	***B***	[6*s*3*p*1*d*], 20	[10*s*7*p*2*d*1*f*], 48	[75]
13	dyall.v2z	***D***	[6*s*1*p*], 9	[10*s*6*p*1*d*], 33	[120]
14	dyall.v3z	***D***	[9*s*2*p*1*d*], 20	[14*s*8*p*2*d*1*f*], 55	[120]
15	cc-pVDZ	***D***	[4*s*1*p*], 7	[9*s*4*p*1*d*], 26	[131]
16	cc-pVTZ	***D***	[5*s*2*p*1*d*], 16	[10*s*5*p*2*d*1*f*], 42	[131]
17	pc-1	***D***	[4*s*1*p*], 7	[7*s*4*p*1*d*], 24	[132]
18	pc-2	***D***	[6*s*2*p*1*d*], 17	[10*s*6*p*2*d*1*f*], 45	[132]
19	pecS-2_ext *	***C***	[12*s*3*p*1*d*], 26	[14*s*7*p*2*d*1*f*], 52	-
20	pcS-2_ext *	***C***	[12*s*3*p*1*d*], 26	[14*s*7*p*2*d*1*f*], 52	-

* pecS-2_ext and pcS-2_ext basis sets are obtained from the pecS-2 and pcS-2 basis sets, respectively, by expanding the tight *s*-region with additional 6 and 4 functions for hydrogen and carbon atoms, respectively, in an even-tempered manner.

## Data Availability

Not applicable.

## Supplementary Materials

The following supporting information can be downloaded at: https://www.mdpi.com/article/10.3390/ijms24076231/s1.
