# Supplementary material for: On the Utmost Importance of the Basis Set Choice for the Calculations of the Relativistic Corrections to NMR Shielding Constants
Source: Int J Mol Sci. 2023 Mar 25;24(7):6231. doi: 10.3390/ijms24076231 (PMC10094374; doi:10.3390/ijms24076231)
Supplement: Supplementary file 1 [file ijms-24-06231-s001.zip › ijms-2303360-supplementary.pdf]

Supplementary Materials

"On the utmost importance of the basis set choice for the calculations of the relativistic corrections to NMR shielding constants"

by Irina L. Rusakova[1,*] and Yuriy Yu. Rusakov[1]

[1]A. E. Favorsky Irkutsk Institute of Chemistry, Siberian Branch of the Russian Academy of Sciences, Favorsky St. 1, Irkutsk 664033, Russia; i-rusakova@bk.ru
*Correspondence: i-rusakova@bk.ru

Table S1. Equilibrium geometries of compounds **1-11** (4c-DFT-PBE0/dyall.av3z).

Compound	Cartesian coordinates, Å			
Hydrogen Iodide, HI (**1**)	I	0.0000000000	0.0000000000	0.0127425617
	H	0.0000000000	0.0000000000	-1.6045326659
Iodomethane, I-CH_3 (**2**)	C	0.0000000000	0.0000000000	-1.9052037645
	I	0.0000000000	0.0000000000	0.2333426888
	H	-1.0338368561	0.0000000000	-2.2324642045
	H	0.5169212092	0.8953306885	-2.2324577549
	H	0.5169212092	-0.8953306885	-2.2324577549
Methylstibine, SbH_2-CH_3 (**3**)	Sb	0.2427384235	0.0000000000	0.0000000000
	H	0.2844357907	1.1986695862	1.2243293174
	H	0.2844357907	1.1986695862	-1.2243293174
	H	-2.3799422027	0.8314551488	0.0000000000
	H	-2.2284480020	-0.7048561422	0.8859933219
	H	-2.2284480020	-0.7048561422	-0.8859933219
	C	-1.9192491803	-0.1527763628	0.0000000000
Methylindium, InH_2-CH_3 (**4**)	In	-0.0005145283	0.2409122207	0.0000000000
	C	-0.0008385651	-1.9159081417	0.0000000000
	H	1.5047072932	1.0994180584	0.0000000000
	H	-1.4837396258	1.1349717045	0.0000000000
	H	-1.0114894873	-2.3205671015	0.0000000000
	H	0.5295843648	-2.2841258719	0.8800071003
	H	0.5295843648	-2.2841258719	-0.8800071003
Methylstannane, SnH_3-CH_3 (**5**)	Sn	0.2288310864	0.0000000000	0.0000000000
	H	0.8180434645	-1.6076173192	0.0000000000
	H	0.8181989878	0.8032132029	1.3925358462
	H	0.8181989878	0.8032132029	-1.3925358462
	H	-2.2871508866	-0.5120010986	0.8865074431
	H	-2.2871508866	-0.5120010986	-0.8865074431
	H	-2.2880756311	1.0236019575	0.0000000000
	C	-1.9162435334	0.0001336337	0.0000000000
TeH-CH_3 (**6**)	Te	0.2279787598	0.0000000000	0.0000000000
	C	-1.9187735817	0.1038266059	0.0000000000
	H	0.2530965313	-1.6628486946	0.0000000000
	H	-2.3106113708	-0.3676840201	0.8959758984
	H	-2.3106113708	-0.3676840201	-0.8959758984
	H	-2.1712809202	1.1619710864	0.0000000000

	C	-0.7685228594	-0.0000169532	0.0000000000
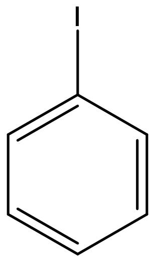	C	-1.4468094324	-1.2063676888	0.0000000000
	C	-2.8324220220	-1.1981783128	0.0000000000
	C	-3.5270815944	-0.0000846469	0.0000000000
	C	-2.8324785367	1.1980422049	0.0000000000
	C	-1.4468661577	1.2063023004	0.0000000000
	I	1.3199726633	0.0000311056	0.0000000000
	H	-0.8950350525	-2.1494079321	0.0000000000
	H	-3.3735811902	-2.1482641458	0.0000000000
1-iodobenzene,	H	-4.6198776189	-0.0001065630	0.0000000000
C_6H_5I (**7**)	H	-3.3736871639	2.1481014416	0.0000000000
	H	-0.8951362240	2.1493693282	0.0000000000
	Sb	0.8331406623	0.0000000000	0.0000000000
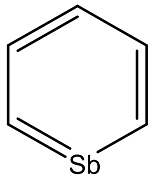	C	-0.5818025971	-1.4704546973	0.0000000000
	C	-1.9388534838	-1.2398630674	0.0000000000
	C	-2.5686937430	-0.0000459670	0.0000000000
	C	-1.9388840722	1.2397908267	0.0000000000
	C	-0.5818917706	1.4705807241	0.0000000000
	H	-0.2351991261	-2.5086251186	0.0000000000
Stibinine,	H	-2.6009414113	-2.1164423580	0.0000000000
C_5H_5Sb (**8**)	H	-3.6628657815	-0.0000752515	0.0000000000
	H	-2.6010569058	2.1163159521	0.0000000000
	H	-0.2352717783	2.5087336737	0.0000000000
	Sn	-0.5919769348	0.0000000000	-0.4937269834
	C	-0.6166383870	0.0000000000	1.5211275730
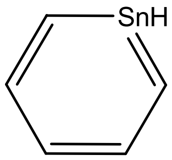	C	0.6150250877	0.0000000000	2.1468967775
	C	1.8857056142	0.0000000000	1.5727812154
	C	2.2226792257	0.0000000000	0.2197602815
	C	1.3859106441	0.0000000000	-0.8795186414
	H	-1.8876428623	0.0000000000	-1.5744714665
	H	-1.5272429346	0.0000000000	2.1227105155
Stannabenzene,	H	0.6010933489	0.0000000000	3.2462057391
C_5H_6Sn (**9**)	H	2.7255977094	0.0000000000	2.2735489173
	H	3.3016594649	0.0000000000	0.0088704459
	H	1.8143427029	0.0000000000	-1.8832942896
	C	0.0000000000	-0.0000733231	0.7156708393
	C	0.0000000000	-1.1972688944	1.4306215071
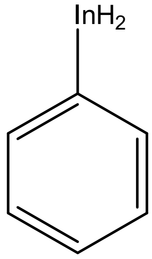	C	0.0000000000	-1.1998059859	2.8162596127
	C	0.0000000000	-0.0000327438	3.5094434109
	C	0.0000000000	1.1997239296	2.8162302585
	C	0.0000000000	1.1971426603	1.4305906223
	In	0.0000000000	0.0000326807	-1.4043502560
	H	0.0000000000	-2.1543882316	0.8965442120
	H	0.0000000000	-2.1477451557	3.3623747102
Phenylindium,	H	0.0000000000	-0.0000130114	4.6031407659
$C_6H_5InH_2$ (**10**)	H	0.0000000000	2.1476797354	3.3623216930
	H	0.0000000000	2.1542441849	0.8964747639
	H	0.0000000000	-1.5131417274	-2.2247727601
	H	0.0000000000	1.5133812216	-2.2244476712

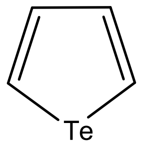 Tellurophene, C_4H_4Te (**11**)	Te	0.6209543895	0.0000000000	0.0000000000
	C	-0.9209043405	-1.3436783192	0.0000000000
	C	-2.1202532510	-0.7090578290	0.0000000000
	C	-2.1202512276	0.7090740986	0.0000000000
	C	-0.9208953451	1.3436772290	0.0000000000
	H	-0.7522618495	-2.4197265668	0.0000000000
	H	-3.0570402827	-1.2756150788	0.0000000000
	H	-3.0570381356	1.2756406779	0.0000000000
	H	-0.7522375066	2.4197211363	0.0000000000

The notation "+ *nk*l" used in the Tables S2-S23 means adding *n* functions of *k*-type (*k* = *s*, *p*, *d*, …) to previous basis set, with *l* designating a particular region within the angular space, namely, *l* = "t" or "d" for t = "tight" and d = "diffuse".

Table S2. The dependence of the α-HALA effect (in ppm) on the proton shielding constants of **1, 3, 4-6** on the basis set used on heavy atom.

Basis set on heavy atom	α-HALA effect on σ(^{1}H)				
	HI (**1**)	SbH_2-CH_3 (**3**)	InH_2-CH_3 (**4**)	SnH_3-CH_3 (**5**)	TeH-CH_3 (**6**)
dyall.v2z	11.5763	1.9620	-5.7555	-0.2145	7.0771
+1st	11.5765	1.9618	-5.7553	-0.2146	7.0770
+2st	11.5764	1.9620	-5.7555	-0.2145	7.0767
+3st	11.5762	1.9620	-5.7553	-0.2146	7.0769
+1sd	11.5800	1.9716	-5.759	-0.2154	7.0828
+2sd	11.5809	1.9667	-5.7595	-0.2168	7.0825
+3sd	11.5809	1.9651	-5.7593	-0.2166	7.0815
+1pt	11.5774	1.9651	-5.7599	-0.2135	7.0831
+2pt	11.5776	1.9651	-5.7600	-0.2135	7.0832
+3pt	11.5777	1.9650	-5.7600	-0.2136	7.0832
+1pd	11.3757	1.9464	-5.7564	-0.2120	6.9978
+2pd	11.3544	1.9428	-5.7539	-0.2105	6.9983
+3pd	11.3470	1.9412	-5.7567	-0.2107	6.9961
+1dt	11.3758	1.9464	-5.7599	-0.2118	6.9962
+2dt	11.3758	1.9464	-5.7599	-0.2118	6.9962
+3dt	11.3758	1.9464	-5.7599	-0.2118	6.9962
+4dt	11.3758	1.9464	-5.7599	-0.2118	6.9962
+5dt	11.3758	1.9464	-5.7599	-0.2118	6.9962
+6dt	11.3758	1.9464	-5.7599	-0.2118	6.9962
+1dd	11.3036	1.9702	-5.7572	-0.2078	6.9740
+2dd	11.3061	1.9669	-5.7546	-0.2097	6.9770
+3dd	11.3066	1.9666	-5.7535	-0.2095	6.9749
+1ft	11.2304	1.9506	-5.7686	-0.2132	6.9340
+2ft	11.2602	1.9597	-5.7703	-0.2127	6.9617
+3ft	11.2733	1.9597	-5.7653	-0.2141	6.9586
+4ft	11.2875	1.9608	-5.7644	-0.2136	6.9661
+5ft	11.2918	1.9618	-5.7635	-0.2136	6.9668

+1fd	11.2481	1.9746	-5.7582	-0.1956	6.9714
+2fd	11.2399	1.9733	-5.7584	-0.1918	6.9717
+3fd	11.2397	1.9728	-5.7585	-0.1913	6.9744
+4fd	11.2397	1.9727	-5.7589	-0.1906	6.9745

Table S3. The dependence of the α-HALA effect (in ppm) on the carbon shielding constants of **2**, **3, 4-6** on the basis set used on heavy atom.

Basis set on heavy atom	α-HALA effect on σ(^{13}C)				
	I-CH_3 (**2**)	SbH_2-CH_3 (**3**)	InH_2-CH_3 (**4**)	SnH_3-CH_3 (**5**)	TeH-CH_3 (**6**)
dyall.v2z	26.4227	8.5135	-8.8456	-1.6108	21.1615
+1st	26.4252	8.514	-8.8476	-1.6105	21.164
+2st	26.4241	8.5129	-8.8451	-1.6107	21.1635
+3st	26.421	8.5134	-8.8478	-1.6107	21.1611
+1sd	26.4195	8.5272	-8.8427	-1.6234	21.1410
+2sd	26.4438	8.5361	-8.8455	-1.6247	21.1203
+3sd	26.4463	8.5328	-8.8457	-1.6248	21.1130
+1pt	26.4488	8.5340	-8.8435	-1.6112	21.1225
+2pt	26.4492	8.5342	-8.8436	-1.6112	21.1228
+3pt	26.4513	8.5347	-8.8436	-1.6109	21.1228
+1pd	25.8051	8.5186	-8.869	-1.6159	20.9318
+2pd	25.8211	8.5270	-8.8752	-1.6170	20.9346
+3pd	25.8354	8.5279	-8.8713	-1.6199	20.9378
+1dt	25.8212	8.5186	-8.8436	-1.6193	20.9379
+2dt	25.8212	8.5186	-8.8435	-1.6194	20.9379
+3dt	25.8212	8.5187	-8.8434	-1.6194	20.9379
+4dt	25.8213	8.5186	-8.8434	-1.6193	20.9379
+5dt	25.8213	8.5186	-8.8434	-1.6193	20.9379
+6dt	25.8212	8.5186	-8.8434	-1.6193	20.9379
+1dd	25.8467	8.6031	-8.8632	-1.5227	20.9668
+2dd	25.8532	8.6188	-8.8679	-1.5261	20.9629
+3dd	25.8535	8.6183	-8.8692	-1.5253	20.9629
+1ft	25.8804	8.6676	-8.9206	-1.5020	20.9552
+2ft	25.8338	8.6592	-8.9148	-1.4982	20.9377
+3ft	25.8263	8.6664	-8.9144	-1.5035	20.9318
+4ft	25.8352	8.6637	-8.9095	-1.5014	20.9411
+5ft	25.8406	8.6622	-8.9135	-1.5021	20.9357
+1fd	25.9216	8.7350	-8.9312	-1.4461	21.0265
+2fd	25.9264	8.7830	-8.9459	-1.4212	21.0524
+3fd	25.9618	8.7855	-8.9478	-1.4205	21.0622
+4fd	25.9646	8.7863	-8.9464	-1.4147	21.0630

Table S4. The dependence of the α-HALA effect (in ppm) on the proton shielding constants of **1, 3, 4-6** on the basis set used on hydrogen atoms.

Basis set on hydrogen atoms	α-HALA effect on σ(^{1}H)				
	HI (**1**)	SbH_2-CH_3 (**3**)	InH_2-CH_3 (**4**)	SnH_3-CH_3 (**5**)	TeH-CH_3 (**6**)
dyall.v2z	11.2397	1.9688	-5.7575	-0.2088	6.9796
+1st	11.6598	2.0508	-5.9522	-0.1995	7.2469
+2st	11.8480	2.0869	-6.0446	-0.1971	7.3644
+3st	11.9102	2.0991	-6.0732	-0.1956	7.4057
+4st	11.9391	2.1046	-6.0875	-0.1953	7.4240
+5st	11.9482	2.1064	-6.0916	-0.1950	7.4298
+1sd	11.9615	2.0890	-6.0744	-0.2067	7.3794
+2sd	11.9594	2.0769	-6.0736	-0.2084	7.3774
+1pt	11.9806	2.0976	-6.0818	-0.2112	7.3949
+2pt	11.9818	2.0980	-6.0825	-0.2112	7.3962
+1pd	12.0126	2.1127	-6.1336	-0.2739	7.4133
+2pd	12.0035	2.1322	-6.1958	-0.2840	7.4239
+3pd	11.9855	2.1167	-6.2039	-0.2808	7.4236
+1dt	12.0105	2.1177	-6.2136	-0.2810	7.3890
+2dt	12.0054	2.1175	-6.2173	-0.2823	7.3986
+1dd	11.9040	2.1045	-6.2187	-0.2806	7.2968
+2dd	11.8892	2.1044	-6.2191	-0.2797	7.2859

Table S5. The dependence of the α-HALA effect (in ppm) on the carbon shielding constants of **2**, **3, 4-6** on the basis set used on carbon atom.

Basis set on caron atom	α-HALA effect on σ(^{13}C)				
	I-CH_3 (**2**)	SbH_2-CH_3 (**3**)	InH_2-CH_3 (**4**)	SnH_3-CH_3 (**5**)	TeH-CH_3 (**6**)
dyall.v2z	25.8532	8.6037	-8.8656	-1.5261	20.9629
+1st	26.6126	8.8889	-8.9604	-1.4220	21.5566
+2st	26.9674	9.0196	-9.0105	-1.3764	21.8408
+3st	27.077	9.0610	-9.0238	-1.3611	21.9291
+4st	27.1295	9.0803	-9.0312	-1.3543	21.9712
+5st	27.1407	9.0847	-9.0324	-1.3528	21.9803
+1sd	27.0723	9.0779	-9.0386	-1.3619	21.9477
+2sd	27.0751	9.0934	-9.038	-1.3719	21.9475
+1pt	27.0023	9.0589	-9.0714	-1.4181	21.8929
+2pt	26.9832	9.0479	-9.0830	-1.4310	21.8773
+1pd	27.0851	9.0964	-9.0897	-1.4076	21.9961
+2pd	27.0692	9.1111	-9.0651	-1.4038	22.0146
+3pd	27.0578	9.1108	-9.0690	-1.4044	22.0316
+1dt	27.0433	9.0966	-9.0855	-1.4165	21.9704
+2dt	26.9384	9.1041	-9.0475	-1.4102	21.9361
+1dd	26.8297	9.1377	-9.1507	-1.4131	21.9754
+2dd	27.0307	9.1364	-9.3301	-1.5084	22.0800

Table S6. Splitting of the α-HALA effect (in ppm) on proton shielding constants of HI (**1**) on SO-HALA and SF-HALA contributions while expanding the basis set used on hydrogen atom.[1]

Basis set on hydrogen atom	SF-HALA	SO-HALA	Total HALA
dyall.v2z	-0.1587	11.4166	11.2579
+1st	-0.1605	11.8420	11.6815
+2st	-0.1628	12.0310	11.8682
+3st	-0.1630	12.0941	11.9311
+4st	-0.1635	12.1232	11.9597
+5st	-0.1635	12.1324	11.9689
+1sd	-0.1561	12.1430	11.9869
+2sd	-0.1566	12.1419	11.9853
+1pt	-0.1289	12.1372	12.0083
+2pt	-0.1281	12.1376	12.0095
+1pd	-0.1456	12.1816	12.0360
+2pd	-0.1328	12.1641	12.0313
+3pd	-0.1348	12.1384	12.0036
+1dt	-0.1491	12.1575	12.0084
+2dt	-0.1504	12.1521	12.0017
+1dd	-0.1572	12.0568	11.8996
+2dd	-0.1577	12.0391	11.8814

[1] The RKB balance is used.

Table S7. Splitting of the α-HALA effect (in ppm) on carbon shielding constant of I-CH_3 (**2**) on SO-HALA and SF-HALA contributions while expanding the basis set used on carbon atom.[1]

Basis set on hydrogen atom	SF-HALA	SO-HALA	Total HALA
dyall.v2z	-3.8011	29.6787	25.8776
+1st	-3.7752	30.4093	26.6341
+2st	-3.7627	30.7540	26.9913
+3st	-3.7591	30.8595	27.1004
+4st	-3.7573	30.9105	27.1532
+5st	-3.7568	30.9212	27.1644
+1sd	-3.7669	30.8532	27.0863
+2sd	-3.7590	30.8474	27.0884
+1pt	-3.7931	30.8083	27.0152
+2pt	-3.8046	30.8007	26.9961
+1pd	-3.7925	30.8899	27.0974

+2pd	-3.7936	30.8773	27.0837
+3pd	-3.8065	30.8755	27.0690
+1dt	-3.7960	30.8505	27.0545
+2dt	-3.8029	30.7332	26.9303
+1dd	-3.8138	30.6491	26.8353
+2dd	-3.8270	30.8720	27.0450

[1] The RKB balance is used.

Table S8. The dependencies of the α-HALA effect (in ppm) from iodine atom on the $\sigma(^1H)$ and of the corresponding $^1J_{FC}(^1H,^{127}I)$ (in Hz) in HI (**1**) on the basis set used on hydrogen atom (at that, dyall.av3z is set on the iodine atom).

Basis set on hydrogen	α-HALA effect on $\sigma(^1H)$	$^1J_{FC}(^1H,^{127}I)$
dyall.aae4z	11.9336	-103.13
aug-cc-pVTZ-J	11.9365	-102.89
ccJ-pVDZ	11.8548	-102.78
ccJ-pVTZ	11.905	-102.71
pcJ-1	11.8695	-103.71
pcJ-2	11.9507	-103.26
pecJ-1	11.8859	-103.05
pecJ-2	11.9389	-102.84
pcS-1	10.1676	-88.61
pcS-2	11.2615	-97.13
pecS-1	10.2744	-88.19
pecS-2	11.2597	-96.87
dyall.v2z	11.2511	-97.87
dyall.v3z	11.7724	-101.60
cc-pVDZ	10.1922	-87.96
cc-pVTZ	10.8867	-93.23
pc-1	10.1607	-88.59
pc-2	11.2578	-97.14
pecS-2_ext	11.9898	-103.37
pcS-2_ext	11.9986	-103.68

Table S9. The dependencies of the α-HALA effect (in ppm) from iodine atom on the $\sigma(^{13}C)$ and of the corresponding $^1J_{FC}(^{13}C,^{127}I)$ (in Hz) in I-CH_3 (**2**) on the basis set used on carbon atom (at that, dyall.av3z is set on the iodine atom).

Basis set on carbon	α-HALA effect on $\sigma(^{13}C)$	$^1J_{FC}(^{13}C,^{127}I)$
dyall.aae4z	26.8483	-139.10
aug-cc-pVTZ-J	27.5976	-139.14
ccJ-pVDZ	27.1733	-138.45
ccJ-pVTZ	27.055	-139.01
pcJ-1	27.1859	-138.29
pcJ-2	26.9246	-138.93
pecJ-1	26.5521	-136.61
pecJ-2	26.7008	-138.25
pcS-1	24.163	-126.13
pcS-2	25.8159	-134.04

pecS-1	21.1931	-115.58
pecS-2	25.9445	-134.24
dyall.v2z	25.9648	-134.39
dyall.v3z	26.9079	-137.93
cc-pVDZ	26.1953	-132.88
cc-pVTZ	26.1072	-134.23
pc-1	24.5776	-125.97
pc-2	25.9025	-134.04
pecS-2_ext	27.3143	-139.37
pcS-2_ext	27.1476	-139.18

Table S10. The dependencies of the HALA effect (in ppm) from Sb atom on $\sigma(^{1}H)$ and $\sigma(^{13}C)$ and the corresponding $J_{FC}(^{1}H,^{121}Sb)$ and $^{1}J_{FC}(^{13}C,^{121}Sb)$ (in Hz) in SbH_2-CH_3 (**3**) on the basis set used on hydrogen and carbon atoms, respectively. Dyall.av3z is set on Sb atom.

Basis set on hydrogen or carbon atom	α-HALA on $\sigma(^{1}H)$	$^{1}J(^{1}H,^{121}Sb)$	β-HALA on $\sigma(^{1}H)^{1}$	$^{2}J(^{1}H,^{121}Sb)^{1}$	α-HALA on $\sigma(^{13}C)$	$^{1}J(^{13}C,^{121}Sb)$
dyall.aae4z	2.2166	63.07	-0.5276	18.58	9.2551	-113.90
aug-cc-pVTZ-J	2.1723	62.78	-0.4910	18.51	9.4335	-114.02
ccJ-pVDZ	2.0945	62.07	-0.4936	18.31	9.2031	-112.38
ccJ-pVTZ	2.1354	62.59	-0.4997	18.42	9.2375	-113.74
pcJ-1	2.1050	61.02	-0.4952	18.38	9.1958	-111.78
pcJ-2	2.1589	62.52	-0.5079	18.48	9.2023	-113.70
pecJ-1	2.1030	62.72	-0.4889	18.29	9.0020	-111.08
pecJ-2	2.1265	62.81	-0.4947	18.45	9.1468	-112.86
pcS-1	1.7761	51.68	-0.4402	15.60	8.0027	-101.85
pcS-2	2.0225	58.63	-0.4858	17.39	8.7158	-109.68
pecS-1	1.8481	53.79	-0.4490	15.40	6.9795	-93.83
pecS-2	2.0261	58.94	-0.4755	17.36	8.7799	-109.85
dyall.v2z	2.0117	59.02	-0.4791	17.39	8.7871	-109.86
dyall.v3z	2.1362	61.67	-0.5027	18.21	9.1419	-112.96
cc-pVDZ	1.7753	53.17	-0.4425	15.55	8.8869	-108.05
cc-pVTZ	1.9323	57.14	-0.4668	16.78	8.8541	-109.84
pc-1	1.7751	51.70	-0.4413	15.64	8.2090	-101.97
pc-2	2.0209	58.63	-0.4862	17.39	8.7576	-109.68
pecS-2_ext	2.1750	62.96	-0.4987	18.53	9.3103	-114.07
pcS-2_ext	2.1707	62.83	-0.5096	18.55	9.2176	-113.90

[1] The values calculated for the methyl protons are averaged.

Table S11. The dependencies of the HALA effect (in ppm) from In atom on σ(^{1}H) and σ(^{13}C) and the corresponding J_{FC}(^{1}H, ^{115}In) and $^1J_{FC}$(^{13}C,^{115}In) (in Hz) in InH_2-CH_3 (**4**) on the basis set used on hydrogen and carbon atom, respectively. Dyall.av3z is set on In atom.

Basis set on hydrogen or carbon atom	α-HALA on σ(^{1}H)[1]	^1J(^{1}H,^{115}In)[1]	β-HALA on σ(^{1}H)[1]	^2J(^{1}H,^{115}In)[1]	α-HALA on σ(^{13}C)	$^1J_{FC}$(^{13}C, ^{115}In)
dyall.aae4z	-6.1783	848.99	-0.4198	-12.53	-9.1346	153.01
aug-cc-pVTZ-J	-6.1593	845.93	-0.4092	-12.45	-8.9209	153.03
ccJ-pVDZ	-6.0979	833.97	-0.4135	-12.05	-9.0084	153.62
ccJ-pVTZ	-6.1389	841.10	-0.4115	-12.40	-9.0344	153.05
pcJ-1	-6.0800	835.20	-0.4076	-11.99	-8.9412	153.80
pcJ-2	-6.1452	844.55	-0.4155	-12.52	-8.9940	152.44
pecJ-1	-6.1323	838.20	-0.4158	-12.36	-8.9722	151.85
pecJ-2	-6.1595	842.61	-0.4108	-12.45	-9.0039	152.64
pcS-1	-5.2699	711.37	-0.3856	-10.16	-8.5950	140.33
pcS-2	-5.8116	793.74	-0.4060	-11.74	-8.8937	147.08
pecS-1	-5.2923	722.79	-0.3806	-11.43	-8.1449	124.82
pecS-2	-5.8181	794.72	-0.3996	-11.76	-8.9289	147.47
dyall.v2z	-5.8133	796.28	-0.4052	-11.85	-8.8488	147.68
dyall.v3z	-6.0633	833.31	-0.4122	-12.36	-9.0387	151.58
cc-pVDZ	-5.3057	713.65	-0.3951	-10.18	-8.6275	146.69
cc-pVTZ	-5.6399	765.33	-0.3963	-11.32	-8.8237	147.71
pc-1	-5.2691	711.12	-0.3879	-10.00	-8.3931	139.66
pc-2	-5.8103	793.72	-0.4066	-11.74	-8.8517	147.09
pecS-2_ext	-6.1725	848.58	-0.4087	-12.63	-9.0706	153.04
pcS-2_ext	-6.1700	848.27	-0.4158	-12.59	-9.0690	152.71

[1] These values are averaged.

Table S12. The dependencies of the HALA effect (in ppm) from Sn atom on σ(^{1}H) and σ(^{13}C) and the corresponding J_{FC}(^{1}H, ^{119}Sn) and $^1J_{FC}$(^{13}C,^{119}Sn) (in Hz) in SnH_3-CH_3 (**5**) on the basis set used on hydrogen and carbon atom, respectively. Dyall.av3z is set on Sn atom.

Basis set on hydrogen or carbon atom	α-HALA on σ(^{1}H)[1]	^1J(^{1}H,^{119}Sn)[1]	β-HALA on σ(^{1}H)[1]	^2J(^{1}H,^{119}Sn)[1]	α-HALA on σ(^{13}C)	$^1J_{FC}$(^{13}C, ^{119}Sn)
dyall.aae4z	-0.1453	-1184.59	-0.4521	19.71	-1.4226	-132.44
aug-cc-pVTZ-J	-0.1730	-1180.23	-0.4209	19.62	-1.0853	-132.33
ccJ-pVDZ	-0.2091	-1165.78	-0.4202	19.11	-1.3045	-133.58
ccJ-pVTZ	-0.2032	-1174.30	-0.4253	19.58	-1.3303	-132.42
pcJ-1	-0.2101	-1166.19	-0.4176	18.94	-1.2980	-134.92
pcJ-2	-0.1941	-1178.10	-0.4277	19.74	-1.3557	-131.86
pecJ-1	-0.2053	-1171.76	-0.4211	19.56	-1.3784	-131.46
pecJ-2	-0.2105	-1176.73	-0.4222	19.64	-1.3678	-132.16
pcS-1	-0.2388	-993.08	-0.3904	16.05	-1.7375	-123.22
pcS-2	-0.2073	-1107.10	-0.4163	18.51	-1.5381	-127.24
pecS-1	-0.2230	-1008.97	-0.3949	17.97	-2.0860	-109.60

pecS-2	-0.2132	-1108.39	-0.4107	18.51	-1.5314	-127.62
dyall.v2z	-0.2072	-1111.04	-0.4147	18.68	-1.4558	-128.03
dyall.v3z	-0.1967	-1162.63	-0.4254	19.45	-1.3793	-131.12
cc-pVDZ	-0.2352	-997.87	-0.3968	16.17	-1.2068	-127.11
cc-pVTZ	-0.2215	-1068.48	-0.4103	17.88	-1.3747	-127.74
pc-1	-0.2371	-992.83	-0.3938	15.84	-1.4414	-122.20
pc-2	-0.2061	-1107.09	-0.4195	18.50	-1.4703	-127.25
pecS-2_ext	-0.1960	-1183.62	-0.4256	19.90	-1.3030	-132.40
pcS-2_ext	-0.1907	-1183.31	-0.4314	19.85	-1.3392	-132.09

[1] These values are averaged.

Table S13. The dependencies of the HALA effect (in ppm) from Te atom on $\sigma(^{1}H)$ and $\sigma(^{13}C)$ and the corresponding $J_{FC}(^{1}H,^{125}Te)$ and $^{1}J_{FC}(^{13}C,^{125}Te)$ (in Hz) in TeH-CH_3 (**6**) on the basis set used on hydrogen and carbon atom, respectively. Dyall.av3z is set on Te atom.

Basis set on hydrogen or carbon atom	α-HALA on $\sigma(^{1}H)$	$^{1}J(^{1}H,^{125}Te)$	β-HALA on $\sigma(^{1}H)$[1]	$^{2}J(^{1}H,^{125}Te)$[1]	α-HALA on $\sigma(^{13}C)$	$^{1}J_{FC}(^{13}C,^{125}Te)$
dyall.aae4z	7.3082	55.91	-0.5381	-24.21	21.7199	198.45
aug-cc-pVTZ-J	7.4957	55.83	-0.5828	-24.13	22.3493	198.56
ccJ-pVDZ	7.3660	55.77	-0.5662	-23.79	22.0091	196.88
ccJ-pVTZ	7.4114	55.63	-0.5718	-23.99	21.9487	198.26
pcJ-1	7.3628	56.99	-0.5695	-23.87	21.9920	196.27
pcJ-2	7.4141	56.12	-0.5849	-24.09	21.8527	198.14
pecJ-1	7.4128	55.89	-0.5590	-23.87	21.5480	194.39
pecJ-2	7.4428	55.80	-0.5686	-24.02	21.7252	197.01
pcS-1	6.2819	48.97	-0.4986	-20.25	19.5025	178.93
pcS-2	6.9771	52.94	-0.5572	-22.66	20.8872	191.15
pecS-1	6.3756	47.34	-0.5186	-20.17	17.1897	164.34
pecS-2	7.0278	52.72	-0.5520	-22.64	21.0264	191.48
dyall.v2z	6.9878	53.16	-0.5482	-22.69	21.0355	191.58
dyall.v3z	7.3253	55.33	-0.5844	-23.75	21.7743	196.79
cc-pVDZ	6.3121	47.71	-0.4995	-20.20	21.2244	189.13
cc-pVTZ	6.7586	50.32	-0.5321	-21.85	21.1343	191.45
pc-1	6.2787	48.93	-0.4981	-20.27	19.8330	178.90
pc-2	6.9739	52.94	-0.5573	-22.66	20.9528	191.15
pecS-2_ext	7.4987	56.23	-0.5825	-24.18	22.1193	198.81
pcS-2_ext	7.4488	56.32	-0.5880	-24.19	21.9585	198.50

[1] These values are averaged.

Table S14. The dependency of the HALA effect (in ppm) from I atom on σ(^{1}H) and σ(^{13}C) in C_6H_5I (**7**) on the basis set used on hydrogen and carbon atom, respectively. Dyall.av3z is set on I atom.

Basis set on hydrogen or carbon atom	α-HALA on σ(^{13}C)	β-HALA on σ(^{13}C)	γ-HALA on σ(^{13}C)	δ-HALA on σ(^{13}C)	γ-HALA on σ(^{1}H)	δ-HALA on σ(^{1}H)	ε-HALA on σ(^{1}H)
dyall.aae4z	31.8004	-0.7185	0.6348	1.2982	-0.3866	0.1623	-0.0852
aug-cc-pVTZ-J	32.5801	-0.8006	0.6502	1.4309	-0.3724	0.1719	-0.1553
ccJ-pVDZ	31.7851	-0.8092	0.6848	1.3252	-0.3737	0.1485	-0.0764
ccJ-pVTZ	31.7297	-0.8513	0.6543	1.2991	-0.3775	0.1538	-0.0849
pcJ-1	32.0128	-0.8531	0.6534	1.3145	-0.3643	0.1519	-0.0770
pcJ-2	31.6822	-0.8751	0.6169	1.2987	-0.3671	0.1669	-0.0879
pecJ-1	31.0189	-0.7964	0.6929	1.3221	-0.3808	0.1442	-0.0760
pecJ-2	31.4156	-0.8536	0.6548	1.3024	-0.3836	0.1578	-0.0745
pcS-1	28.6056	-0.8245	0.5429	1.1417	-0.2924	0.1291	-0.0625
pcS-2	30.3237	-0.9137	0.5550	1.2270	-0.3397	0.1583	-0.0795
pecS-1	26.1807	-0.7818	0.4240	1.0007	-0.2874	0.1364	-0.0839
pecS-2	30.2980	-0.9239	0.5651	1.2387	-0.3477	0.1546	-0.0728
dyall.v2z	30.6829	-0.7980	0.6118	1.2628	-0.3390	0.1537	-0.0797
dyall.v3z	31.4477	-0.8956	0.5959	1.2618	-0.3716	0.1617	-0.0811
cc-pVDZ	30.6501	-0.6775	0.7504	1.3440	-0.2999	0.1304	-0.0584
cc-pVTZ	30.7067	-0.7262	0.6851	1.2697	-0.3339	0.1443	-0.0690
pc-1	28.9778	-0.7322	0.6370	1.2437	-0.2917	0.1325	-0.0596
pc-2	30.4226	-0.8768	0.5930	1.2666	-0.3406	0.1552	-0.0838
pecS-2_ext	31.8442	-0.8723	0.6252	1.3120	-0.3793	0.1660	-0.0799
pcS-2_ext	31.8387	-0.8706	0.6184	1.2997	-0.3706	0.1671	-0.0896

[1] These values are averaged.

Table S15. The dependencies of the SSCCs $J_{FC}(^{1}H, ^{127}I)$ and $^{1}J_{FC}(^{13}C, ^{127}I)$ (in Hz) in C_6H_5I (**7**) on the basis set used on hydrogen and carbon atom, respectively. Dyall.av3z is set on I atom.

Basis set on hydrogen or carbon atom	$^{1}J_{FC}(^{13}C, ^{127}I)$	$^{2}J_{FC}(^{13}C, ^{127}I)$	$^{3}J_{FC}(^{13}C, ^{127}I)$	$^{4}J_{FC}(^{13}C, ^{127}I)$	$^{3}J_{FC}(^{1}H, ^{127}I)$	$^{4}J_{FC}(^{1}H, ^{127}I)$	$^{5}J_{FC}(^{1}H, ^{127}I)$
dyall.aae4z	-184.66	12.32	0.76	-0.86	9.65	1.21	2.88
aug-cc-pVTZ-J	-184.75	12.31	0.76	-0.85	9.62	1.21	2.88
ccJ-pVDZ	-184.11	12.04	0.72	-0.85	9.50	1.20	2.87
ccJ-pVTZ	-184.29	12.32	0.80	-0.86	9.62	1.21	2.87
pcJ-1	-184.46	11.91	0.57	-0.82	9.36	1.16	2.86
pcJ-2	-184.44	12.33	0.74	-0.85	9.58	1.20	2.87
pecJ-1	-180.90	12.17	0.83	-0.88	9.72	1.25	2.88
pecJ-2	-183.27	12.29	0.83	-0.86	9.66	1.22	2.88
pcS-1	-168.19	10.88	0.53	-0.75	7.95	0.98	2.43
pcS-2	-177.93	11.90	0.71	-0.82	9.01	1.13	2.69
pecS-1	-157.89	9.64	0.56	-0.59	7.95	0.93	2.32

pecS-2	-177.98	11.89	0.72	-0.83	9.04	1.13	2.70
dyall.v2z	-178.61	11.95	0.66	-0.83	9.00	1.11	2.70
dyall.v3z	-183.02	12.21	0.75	-0.85	9.47	1.19	2.83
cc-pVDZ	-176.79	11.52	0.69	-0.81	8.17	1.04	2.49
cc-pVTZ	-177.93	11.88	0.77	-0.83	8.77	1.11	2.62
pc-1	-168.08	10.79	0.51	-0.74	7.96	1.00	2.45
pc-2	-177.93	11.89	0.71	-0.82	9.01	1.13	2.70
pecS-2_ext	-184.80	12.34	0.74	-0.86	9.65	1.20	2.88
pcS-2_ext	-184.77	12.35	0.74	-0.86	9.62	1.20	2.88

Table S16. The dependency of the HALA effect (in ppm) from Sb atom on σ(^{1}H) and σ(^{13}C) in C_5H_5Sb (**8**) on the basis set used on hydrogen and carbon atom, respectively. Dyall.av3z is set on Sb atom.

Basis set on hydrogen or carbon atom	α-HALA on σ(^{13}C)	β-HALA on σ(^{13}C)	γ-HALA on σ(^{13}C)	β-HALA on σ(^{1}H)	γ-HALA on σ(^{1}H)	δ-HALA on σ(^{1}H)
dyall.aae4z	-1.2692	1.0172	-7.1472	-1.0931	-0.2688	-0.3414
aug-cc-pVTZ-J	-0.6004	1.0358	-6.9200	-1.0800	-0.2234	-0.3207
ccJ-pVDZ	-1.1068	0.9516	-6.9503	-0.9846	-0.2245	-0.3135
ccJ-pVTZ	-1.0491	0.9961	-7.1219	-1.0437	-0.2405	-0.3265
pcJ-1	-1.1348	0.9508	-6.9452	-0.9815	-0.2318	-0.3106
pcJ-2	-1.0738	0.9915	-7.1691	-1.0607	-0.2428	-0.3217
pecJ-1	-0.9756	0.9212	-6.9075	-0.9839	-0.2169	-0.3119
pecJ-2	-1.0340	0.9711	-7.1457	-1.0401	-0.2267	-0.3198
pcS-1	-1.3667	0.8517	-6.5689	-0.8811	-0.1896	-0.2887
pcS-2	-1.2316	0.9511	-6.9349	-1.0193	-0.2254	-0.3134
pecS-1	-2.4379	0.9357	-6.3675	-0.9091	-0.2540	-0.2963
pecS-2	-1.2881	0.9390	-6.9075	-1.0147	-0.2286	-0.3022
dyall.v2z	-1.2059	0.9925	-6.8117	-0.9664	-0.2194	-0.3034
dyall.v3z	-1.2028	1.0349	-7.0369	-1.0317	-0.2466	-0.3131
cc-pVDZ	-0.9259	0.9788	-6.5493	-0.8835	-0.1832	-0.2889
cc-pVTZ	-1.0287	0.9717	-6.8585	-0.9799	-0.2102	-0.3072
pc-1	-1.1002	0.9257	-6.3316	-0.8788	-0.1872	-0.2843
pc-2	-1.1613	0.9817	-6.8749	-1.0193	-0.2280	-0.3159
pecS-2_ext	-1.1810	0.9923	-7.0527	-1.0587	-0.2452	-0.3086
pcS-2_ext	-1.1587	0.9900	-7.0630	-1.0668	-0.2447	-0.3226

Table S17. The dependencies of the SSCCs $J_{FC}(^1H, ^{121}Sb)$ and $^1J_{FC}(^{13}C, ^{121}Sb)$ (in Hz) in C_5H_5Sb (**8**) on the basis set used on hydrogen and carbon atom, respectively. Dyall.av3z is set on Sb atom.

Basis set on hydrogen or carbon atom	$^1J_{FC}(^{13}C, ^{121}Sb)$	$^2J_{FC}(^{13}C, ^{121}Sb)$	$^3J_{FC}(^{13}C, ^{121}Sb)$	$^2J_{FC}(^1H, ^{121}Sb)$	$^3J_{FC}(^1H, ^{121}Sb)$	$^4J_{FC}(^1H, ^{121}Sb)$
dyall.aae4z	-159.34	-7.19	3.84	73.12	5.15	1.33
aug-cc-pVTZ-J	-159.58	-7.22	3.83	72.87	5.13	1.33
ccJ-pVDZ	-157.80	-7.43	3.79	71.96	4.96	1.39
ccJ-pVTZ	-158.97	-7.24	3.86	72.53	5.11	1.35
pcJ-1	-157.26	-7.62	3.64	71.64	4.89	1.36
pcJ-2	-158.94	-7.22	3.82	72.68	5.09	1.34
pecJ-1	-155.48	-7.36	3.87	72.52	5.05	1.41
pecJ-2	-157.93	-7.20	3.89	72.71	5.11	1.37
pcS-1	-143.31	-6.94	3.34	60.90	4.15	1.16
pcS-2	-153.33	-6.97	3.70	68.35	4.79	1.26
pecS-1	-133.14	-6.22	3.71	61.49	4.23	1.13
pecS-2	-153.29	-6.92	3.74	68.36	4.80	1.26
dyall.v2z	-153.84	-6.98	3.61	68.26	4.72	1.28
dyall.v3z	-157.85	-7.16	3.80	71.69	5.06	1.31
cc-pVDZ	-151.76	-7.25	3.84	61.44	4.27	1.18
cc-pVTZ	-153.55	-7.01	3.76	66.07	4.67	1.23
pc-1	-143.56	-7.00	3.38	61.02	4.15	1.16
pc-2	-153.34	-6.97	3.70	68.36	4.79	1.26
pecS-2_ext	-159.17	-7.19	3.88	73.00	5.12	1.35
pcS-2_ext	-159.23	-7.24	3.84	72.99	5.11	1.34

Table S18. The dependency of the HALA effect (in ppm) from Sn atom on $\sigma(^1H)$ and $\sigma(^{13}C)$ in C_5H_6Sn (**9**) on the basis set used on hydrogen and carbon atom, respectively. Dyall.av3z is set on Sn atom.

Basis set on hydrogen or carbon atom	α-HALA on $\sigma(^{13}C)$	β-HALA on $\sigma(^{13}C)$	γ-HALA on $\sigma(^{13}C)$	α -HALA on $\sigma(^1H)$	β-HALA on $\sigma(^1H)$	γ-HALA on $\sigma(^1H)$	δ-HALA on $\sigma(^1H)$
dyall.aae4z	-4.9609	0.7002	-0.7321	-2.7287	-0.9199	-0.2348	-0.0889
aug-cc-pVTZ-J	-4.0240	0.9439	-0.7460	-2.6333	-0.8862	-0.1717	-0.0678
ccJ-pVDZ	-4.5409	0.8750	-0.7810	-2.5626	-0.7811	-0.1717	-0.0727
ccJ-pVTZ	-4.5954	0.8658	-0.8153	-2.6202	-0.8177	-0.1838	-0.0749
pcJ-1	-4.5672	0.8625	-0.7814	-2.5798	-0.7739	-0.1754	-0.0735
pcJ-2	-4.6130	0.8665	-0.8155	-2.6465	-0.8213	-0.1853	-0.0717
pecJ-1	-4.4563	0.8771	-0.7740	-2.5618	-0.7797	-0.1727	-0.0733
pecJ-2	-4.5973	0.8542	-0.8341	-2.6228	-0.8186	-0.1758	-0.0679
pcS-1	-4.4952	0.6992	-0.7532	-2.2850	-0.6802	-0.1431	-0.0622
pcS-2	-4.5835	0.8087	-0.7715	-2.5231	-0.7825	-0.1717	-0.0677
pecS-1	-4.5887	0.7216	-0.6717	-2.3205	-0.6668	-0.1596	-0.0706

pecS-2	-4.6166	0.7827	-0.7718	-2.4950	-0.7840	-0.1753	-0.0660
dyall.v2z	-4.4988	0.8548	-0.7357	-2.5019	-0.7505	-0.1689	-0.0642
dyall.v3z	-4.6259	0.8760	-0.7732	-2.6219	-0.8043	-0.1851	-0.0653
cc-pVDZ	-4.2387	0.9017	-0.6883	-2.2678	-0.6881	-0.1399	-0.0593
cc-pVTZ	-4.4205	0.8451	-0.7646	-2.4363	-0.7577	-0.1572	-0.0617
pc-1	-4.2386	0.8031	-0.6666	-2.2801	-0.6784	-0.1402	-0.0576
pc-2	-4.5175	0.8436	-0.7407	-2.5212	-0.7843	-0.1744	-0.0700
pecS-2_ext	-4.6272	0.8433	-0.7841	-2.6233	-0.8261	-0.1909	-0.0721
pcS-2_ext	-4.6095	0.8723	-0.7692	-2.6516	-0.8260	-0.1871	-0.0717

Table S19. The dependencies of the SSCCs $J_{FC}(^1H, ^{119}Sb)$ and $^1J_{FC}(^{13}C, ^{119}Sb)$ (in Hz) in C_5H_6Sn (**9**) on the basis set used on hydrogen and carbon atom, respectively. Dyall.av3z is set on Sn atom.

Basis set on hydrogen or carbon atom	$^1J_{FC}(^{13}C, ^{119}Sn)$	$^2J_{FC}(^{13}C, ^{119}Sn)$	$^3J_{FC}(^{13}C, ^{119}Sn)$	$^1J_{FC}(^1H, ^{119}Sn)$	$^2J_{FC}(^1H, ^{119}Sn)$	$^3J_{FC}(^1H, ^{119}Sn)$	$^4J_{FC}(^1H, ^{119}Sn)$
dyall.aae4z	-347.59	4.70	-130.98	-1615.44	-90.47	-126.73	11.42
aug-cc-pVTZ-J	-347.13	4.59	-130.89	-1610.34	-90.16	-126.49	11.38
ccJ-pVDZ	-353.03	4.54	-129.36	-1595.30	-88.64	-124.29	11.44
ccJ-pVTZ	-348.78	4.56	-130.71	-1601.05	-89.51	-125.63	11.42
pcJ-1	-352.24	4.39	-128.53	-1601.75	-87.97	-124.13	11.35
pcJ-2	-347.30	4.59	-130.70	-1606.38	-89.75	-126.05	11.37
pecJ-1	-347.67	4.67	-128.60	-1600.14	-89.04	-125.17	11.61
pecJ-2	-347.17	4.61	-130.12	-1604.31	-89.73	-125.95	11.49
pcS-1	-321.53	4.12	-117.40	-1363.77	-74.81	-105.63	9.67
pcS-2	-335.06	4.44	-126.12	-1509.55	-84.42	-118.48	10.68
pecS-1	-304.68	4.05	-104.44	-1378.91	-75.74	-106.10	9.66
pecS-2	-336.16	4.63	-126.28	-1511.47	-84.43	-118.52	10.69
dyall.v2z	-338.32	4.88	-126.62	-1520.16	-84.02	-118.23	10.75
dyall.v3z	-344.58	4.64	-129.78	-1585.34	-88.55	-124.45	11.22
cc-pVDZ	-337.23	4.42	-124.14	-1365.14	-76.10	-106.57	9.75
cc-pVTZ	-336.40	4.37	-126.25	-1457.00	-81.59	-114.46	10.40
pc-1	-319.40	3.87	-117.08	-1363.38	-75.31	-105.95	9.65
pc-2	-335.05	4.43	-126.10	-1509.52	-84.44	-118.50	10.68
pecS-2_ext	-348.93	4.80	-131.07	-1614.06	-90.14	-126.62	11.44
pcS-2_ext	-347.90	4.60	-130.93	-1613.47	-90.13	-126.60	11.42

Table S20. The dependency of the HALA effect (in ppm) from In atom on σ(^{1}H) and σ(^{13}C) in $C_6H_5InH_2$ (**10**) on the basis set used on hydrogen and carbon atom, respectively. Dyall.av3z is set on In atom.

Basis set on hydrogen or carbon atom	α-HALA on σ(^{13}C)	β-HALA on σ(^{13}C)	γ-HALA on σ(^{13}C)	δ-HALA on σ(^{13}C)	α -HALA on σ(^{1}H)	β-HALA on σ(^{1}H)	γ-HALA on σ(^{1}H)	δ-HALA on σ(^{1}H)
dyall.aae4z	-10.0811	0.7542	0.6423	1.1499	-5.8460	-0.1192	-0.1066	-0.0522

aug-cc-pVTZ-J	-9.8215	0.8054	0.6874	1.2481	-5.9655	-0.1134	-0.1202	-0.0889
ccJ-pVDZ	-8.4431	0.5524	0.5369	1.0690	-5.4138	-0.1175	-0.0856	-0.0354
ccJ-pVTZ	-8.4676	0.5401	0.5330	1.0661	-5.4776	-0.1206	-0.0864	-0.0370
pcJ-1	-9.5902	0.7941	0.6382	1.1393	-5.8345	-0.1172	-0.0884	-0.0350
pcJ-2	-9.6015	0.7981	0.6507	1.1505	-5.9148	-0.1221	-0.0910	-0.0313
pecJ-1	-9.5279	0.7929	0.6313	1.1369	-5.8776	-0.1206	-0.0927	-0.0389
pecJ-2	-9.6384	0.7951	0.6418	1.1485	-5.9187	-0.1196	-0.0883	-0.0306
pcS-1	-8.9347	0.7107	0.5284	0.9951	-5.0595	-0.1021	-0.0772	-0.0298
pcS-2	-9.3996	0.7097	0.5757	1.0896	-5.5944	-0.1157	-0.0842	-0.0261
pecS-1	-8.5659	0.6855	0.5335	0.9567	-5.0829	-0.1057	-0.0758	-0.0309
pecS-2	-9.4725	0.6900	0.5701	1.0928	-5.5958	-0.1160	-0.0827	-0.0283
dyall.v2z	-9.3273	0.8404	0.6381	1.1027	-5.5828	-0.1144	-0.0854	-0.0323
dyall.v3z	-9.6300	0.7503	0.5872	1.1039	-5.8413	-0.1187	-0.0859	-0.0239
cc-pVDZ	-8.0377	0.5909	0.5797	1.0904	-4.7189	-0.1013	-0.0745	-0.0297
cc-pVTZ	-8.1519	0.5728	0.5481	1.0662	-5.0367	-0.1102	-0.0802	-0.0329
pc-1	-8.7710	0.7709	0.6196	1.0833	-5.0574	-0.0995	-0.0752	-0.0267
pc-2	-9.3482	0.7434	0.6136	1.1276	-5.5930	-0.1159	-0.0875	-0.0306
pecS-2_ext	-9.6597	0.8046	0.6421	1.1338	-5.9348	-0.1222	-0.0885	-0.0323
pcS-2_ext	-9.6433	0.7940	0.6266	1.1417	-5.9395	-0.1222	-0.0914	-0.0320

Table S21. The dependencies of the SSCCs $J_{FC}(^{1}H, ^{115}In)$ and $^{1}J_{FC}(^{13}C, ^{115}In)$ (in Hz) in $C_6H_5InH_2$ (**10**) on the basis set used on hydrogen and carbon atom, respectively. Dyall.av3z is set on Sn atom.

Basis set on hydrogen or carbon atom	$^{1}J_{FC}(^{13}C, ^{115}In)$	$^{2}J_{FC}(^{13}C, ^{115}In)$	$^{3}J_{FC}(^{13}C, ^{115}In)$	$^{4}J_{FC}(^{13}C, ^{115}In)$	$^{1}J_{FC}(^{1}H, ^{115}In)$	$^{3}J_{FC}(^{1}H, ^{115}In)$	$^{4}J_{FC}(^{1}H, ^{115}In)$	$^{5}J_{FC}(^{1}H, ^{115}In)$
dyall.aae4z	281.29	30.59	25.52	-3.01	870.13	32.26	6.36	2.52
aug-cc-pVTZ-J	281.29	30.65	25.55	-3.02	867.13	32.13	6.36	2.52
ccJ-pVDZ	282.18	30.28	25.54	-3.03	856.02	31.95	6.31	2.59
ccJ-pVTZ	281.57	30.61	25.60	-3.03	861.56	32.07	6.34	2.53
pcJ-1	281.85	29.88	25.30	-3.02	859.46	31.68	6.03	2.76
pcJ-2	280.51	30.48	25.49	-3.01	865.73	32.10	6.33	2.53
pecJ-1	278.29	30.05	25.43	-3.01	857.99	32.37	6.38	2.58
pecJ-2	280.26	30.44	25.50	-3.02	863.08	32.21	6.37	2.56
pcS-1	257.20	27.25	23.06	-2.76	731.88	26.95	5.13	2.34
pcS-2	270.63	29.41	24.59	-2.91	813.64	30.19	5.95	2.38
pecS-1	243.15	26.70	20.56	-2.15	742.07	27.03	5.00	2.13
pecS-2	271.68	29.45	24.50	-2.90	814.28	30.22	5.92	2.39
dyall.v2z	272.30	29.37	24.46	-2.89	854.75	31.79	6.16	2.51
dyall.v3z	278.80	30.31	25.29	-2.99	853.98	31.68	6.24	2.49
cc-pVDZ	270.65	29.21	24.61	-2.92	731.94	27.33	5.45	2.25
cc-pVTZ	271.74	29.61	24.74	-2.93	783.88	29.20	5.78	2.31
pc-1	256.27	27.39	23.13	-2.75	731.66	26.91	5.21	2.35
pc-2	270.61	29.42	24.59	-2.91	813.63	30.18	5.95	2.38
pecS-2_ext	282.04	30.57	25.43	-3.01	869.50	32.28	6.33	2.56
pcS-2_ext	281.02	30.54	25.53	-3.02	869.50	32.24	6.36	2.54

Table S22. The dependency of the HALA effect (in ppm) from Te atom on σ(^{1}H) and σ(^{13}C) in C_4H_4Te (**11**) on the basis set used on hydrogen and carbon atom, respectively. Dyall.av3z is set on Te atom.

Basis set on hydrogen or carbon atom	α-HALA on σ(^{13}C)	β-HALA on σ(^{13}C)	β-HALA on σ(^{1}H)	γ-HALA on σ(^{1}H)
dyall.aae4z	8.5279	-5.2057	-1.2085	-0.8298
aug-cc-pVTZ-J	8.8382	-5.2318	-1.2374	-0.6279
ccJ-pVDZ	8.7024	-5.2509	-1.1885	-0.6499
ccJ-pVTZ	8.6169	-5.3112	-1.2200	-0.6672
pcJ-1	8.6256	-5.2465	-1.1917	-0.6444
pcJ-2	8.5143	-5.3173	-1.2314	-0.6737
pecJ-1	8.6926	-5.2046	-1.1824	-0.6557
pecJ-2	8.5877	-5.3225	-1.2183	-0.6561
pcS-1	7.3542	-4.8419	-1.0555	-0.5626
pcS-2	7.9685	-5.1309	-1.1775	-0.6403
pecS-1	6.0521	-4.3191	-1.0501	-0.5853
pecS-2	8.0251	-5.1523	-1.1779	-0.6579
dyall.v2z	7.5306	-4.8291	-1.0845	-0.5736
dyall.v3z	8.5249	-5.2449	-1.1917	-0.6961
cc-pVDZ	7.8836	-4.7433	-0.9995	-0.5142
cc-pVTZ	8.3487	-5.0827	-1.1180	-0.6397
pc-1	7.6867	-4.7292	-1.0539	-0.5578
pc-2	8.0570	-5.0874	-1.1772	-0.6409
pecS-2_ext	8.5773	-5.3063	-1.2382	-0.6954
pcS-2_ext	8.5305	-5.2821	-1.2367	-0.6762

Table S23. The dependencies of the SSCCs J_{FC}(^{1}H, ^{125}Te) and $^{1}J_{FC}$(^{13}C, ^{125}Te) (in Hz) in C_4H_4Te (**11**) on the basis set used on hydrogen and carbon atom, respectively. Dyall.av3z is set on Sn atom.

Basis set on hydrogen or carbon atom	$^{1}J_{FC}$(^{13}C, ^{125}Te)	$^{2}J_{FC}$(^{13}C, ^{125}Te)	$^{2}J_{FC}$(^{1}H, ^{125}Te)	$^{3}J_{FC}$(^{1}H, ^{125}Te)
dyall.aae4z	204.20	-0.41	-106.19	-19.94
aug-cc-pVTZ-J	204.47	-0.38	-105.85	-19.89
ccJ-pVDZ	202.81	-0.13	-104.31	-19.41
ccJ-pVTZ	203.73	-0.48	-105.33	-19.76
pcJ-1	202.29	0.24	-103.79	-19.30
pcJ-2	203.72	-0.42	-105.56	-19.79
pecJ-1	199.34	-0.46	-105.10	-19.65
pecJ-2	202.34	-0.57	-105.57	-19.81
pcS-1	184.36	0.21	-88.25	-16.41
pcS-2	196.52	-0.41	-99.26	-18.61
pecS-1	173.56	0.59	-89.32	-16.40
pecS-2	196.62	-0.46	-99.26	-18.62
dyall.v2z	197.25	-0.33	-99.19	-18.58
dyall.v3z	202.37	-0.41	-104.14	-19.58

cc-pVDZ	195.20	-0.07	-89.24	-16.72
cc-pVTZ	196.77	-0.45	-95.98	-18.04
pc-1	184.64	0.29	-88.43	-16.45
pc-2	196.54	-0.40	-99.27	-18.61
pecS-2_ext	204.16	-0.48	-106.04	-19.88
pcS-2_ext	204.09	-0.42	-106.01	-19.88
